# Integrated bioinformatics analysis elucidates granulosa cell whole-transcriptome landscape of PCOS in China

**DOI:** 10.1186/s13048-023-01223-0

**Published:** 2023-08-03

**Authors:** Qingfang Li, Yimiao Sang, Qingqing Chen, Bingru Ye, Xiaoqian Zhou, Yimin Zhu

**Affiliations:** 1 School of Medicine, Women’s Hospital, Zhejiang University, 1 Xueshi Road, Shangcheng District, Hangzhou, 310006 China; 2 Key Laboratory of Reproductive Genetics, Ministry of Education Zhejiang University, Hangzhou, 310006 China; 3 Department of Reproductive Endocrinology, Women’s Hospital, Zhejiang University School of Medicine, Hangzhou, Zhejiang 310006 China

**Keywords:** PCOS (polycystic ovarian syndrome), Bioinformatics analysis, GEO database, ceRNA network, Correlation analysis

## Abstract

**Background:**

Polycystic ovary syndrome (PCOS) is a common reproductive, neuroendocrine, and metabolic disorder in women of reproductive age that affects up to 5–10% of women of reproductive age. The aetiology of follicle development arrest and critical issues regarding the abnormal follicular development in PCOS remain unclear. The present study aims to systematically evaluate granulosa cell whole-transcriptome sequencing data to gain more insights into the transcriptomic landscape and molecular mechanism of PCOS in China.

**Methods:**

In the present study, the microarray datasets GSE138518, GSE168404, GSE193123, GSE138572, GSE95728, and GSE145296 were downloaded from the Gene Expression Omnibus (GEO) database. Subsequently, differential expression analysis was performed on the PCOS and control groups, followed by functional interaction prediction analysis to investigate gene-regulatory circuits in PCOS. Finally, hub genes and their associated ncRNAs were validated by qPCR in human-luteinized granulosa (hGL) cells and were correlated with the clinical characteristics of the patients.

**Results:**

A total of 200 differentially expressed mRNAs, 3 differentially expressed miRNAs, 52 differentially expressed lncRNAs, and 66 differentially expressed circRNAs were found in PCOS samples compared with controls. GO and KEGG enrichment analyses indicated that the DEGs were mostly enriched in phospholipid metabolic processes, steroid biosynthesis and inflammation related pathways. In addition, the upregulated miRNA hsa-miR-205-5p was significantly enriched in the ceRNA network, and two hub genes, MVD and PNPLA3, were regulated by hsa-miR-205-5p, which means that hsa-miR-205-5p may play a fundamental role in the pathogenesis of PCOS. We also found that MVD and PNPLA3 were related to metabolic processes and ovarian steroidogenesis, which may be the cause of the follicle development arrest in PCOS patients.

**Conclusions:**

In summary, we systematically constructed a ceRNA network depicting the interactions between the ncRNAs and the hub genes in PCOS and control subjects and correlated the hub genes with the clinical characteristics of the patients, which provides valuable insights into the granulosa cell whole-transcriptome landscape of PCOS in China.

**Supplementary Information:**

The online version contains supplementary material available at 10.1186/s13048-023-01223-0.

## Introduction

Polycystic ovary syndrome (PCOS) is a common reproductive, neuroendocrine, and metabolic disorder in women of reproductive age that affects up to 5–10% of reproductive aged women [1]. Its main clinical manifestations are ovulatory dysfunction, hyperandrogenemia, and polycystic ovaries, which can lead to infertility [2, 3]. Additional clinical features include metabolic abnormalities such as insulin resistance, obesity and type 2 diabetes (T2D) [4]. Although research on the aetiology of follicle development arrest in patients with PCOS has continuously emerged in recent years, critical issues regarding abnormal follicular development in PCOS and the precise network regulation mechanism remain unclear.

Granulosa cells (GCs) are an important cell type surrounding follicles that can interfere with follicle maturation and ovulation [5]. Several studies have shown that GC dysfunction is associated with the disruption of follicle development, such as excessive follicular recruitment, obstruction of dominant follicular selection, follicular atresia, and anovulation and metabolic disorder in PCOS [6–10]. Folliculogenesis and steroidogenesis rely on oocyte-GC crosstalk, which can provide oocytes with nutrients and the removal of waste. The communication between oocytes and GCs is critical for normal follicular development and the secretion of steroid hormones by GCs [11]. Moreover, studies have suggested that the metabolic process of GCs has an effect on follicular development [12, 13]. Until now, how GC dysfunction leads to the occurrence of PCOS has not been elucidated.

In recent years, an increasing number of studies have performed transcriptome sequencing of GCs to explore the pathogenesis of PCOS [14–17]. Competing endogenous RNAs (ceRNAs), natural decoys that compete for a common pool of microRNAs (miRNAs), represent a novel layer of gene regulation by systematically functionalizing miRNA response element (MRE)-harboring noncoding RNAs, such as long noncoding RNAs (lncRNAs), pseudogenes, and circular RNAs (circRNAs), and forming complex miRNA-mediated ceRNA networks [18–21]. Perturbation of the ceRNA crosstalk balance of cellular processes and functions leads to diseases such as PCOS [22–24]. Recently, a study reported that lncXIST inhibited human granulosa-like tumor cell viability and induced apoptosis by increasing the expression of Bcl2-like protein 11 (BCL2L11) via the sponging of miR-30c-5p by ceRNA [23]. However, few studies have utilized GCs whole-transcriptome sequencing strategies, which allow accurate examination of global gene expression profiles, to elucidate the GCs transcriptome characteristics of PCOS in China.

In this study, we investigated the whole-transcriptome profiles in GCs of PCOS patients by using GEO datasets. Subsequently, differential mRNA, miRNA, lncRNA and circRNA expression analyses were performed between the control groups and PCOS groups, followed by functional interaction prediction analysis. The results showed that PNPLA3, MVD, MMP9, LCK, NCF1, OSM, C3, MLXIPL and TREM1 were closely related to the onset of PCOS. In addition, miR-205-5p, miR-210-5p, and miR-144-5p and their associated lncRNAs and circRNAs were also involved in the progression of PCOS. This study uncovered a reliable molecular basis of PCOS initiation and progression, and provided clues to investigate the onset and development of PCOS.

## Methods

### Microarray data

The National Center for Biotechnology Information (NCBI)’s Gene Expression Omnibus (GEO) repository (https://www.ncbi.nlm.nih.gov/geo/) was searched for gene expression profile datasets of GCs from women with or without PCOS. Gene expression datasets for 6 PCOS studies were downloaded (GSE138518, GSE168404, GSE193123, GSE138572, GSE95728, and GSE145296) from Gene Expression Omnibus (GEO, https://www.ncbi.nlm.nih.gov/geo/), including 34 control (control group), and 34 PCOS patients (case group) from China, as shown in Table 1. Background correction, and quantile normalization, were performed by RMA normalization.

**Table 1:** The basic backgrounds of the datasets included

	Dataset ID	Platform	PCOS	Normal
mRNA	GSE138518	GPL11154	3	3
mRNA	GSE168404	GPL16791	5	5
mRNA	GSE193123	GPL24676	3	3
miRNA	GSE138572	GPL11154	5	5
miRNA	GSE168404	GPL16791	5	5
lncRNA	GSE95728	GPL16956	7	7
circRNA	GSE145296	GPL28148	6	6

### Identification of differentially expressed mRNAs and noncoding RNAs

Differential gene analysis was performed by edgeR and the limma package. We defined RNAs with a *P* value < 0.05 and |log FC| > 0.5 as being differentially expressed. Overlapping differentially expressed mRNAs were identified in at least two mRNA datasets (GSE138518, GSE168404, and GSE193123). Overlapping differentially expressed miRNAs were identified in two miRNA datasets (GSE138572 and GSE168404). Furthermore, we identified differentially expressed lncRNAs and circRNAs in the lncRNA dataset (GSE95728) and circRNA dataset (GSE145296).

### Protein-Protein Interaction (PPI) network, module extraction and hub genes identification

To further investigate the function of dif-mRNAs at the protein level, we constructed a PPI network using the STRING database (https://string-db.org/) and visualized it by Cytoscape. Interactions with a combined score ≥ 0.4 were considered statistically significant. Then, to identify highly interacting hub mRNA clustering, we established “Molecular Complex Detection” (MCODE), a clustering algorithm that identifies locally densely connected regions in a large PPI network based on node-weighting arithmetic with degree cut-off score = 2, k-core = 2 and max depth = 100. The cytoHubba app in Cytoscape was used to disclose the hub genes in the PPI network. In the whole PPI network, the top 50 hub genes ranked by maximal clique centrality (MCC) were obtained.

### Functional annotation and enrichment analysis

Gene Ontology (GO) annotation and Kyoto Encyclopedia of Genes and Genomes (KEGG) pathway enrichment analyses were conducted to investigate the roles of the differentially expressed mRNAs. GO enrichment analysis and KEGG enrichment analysis were carried out using clusterProfiler. A GO tree was constructed to summarize the affected functions. The mutual regulatory relationships between enriched KEGG pathways were illustrated by Pathway-Act networks.

### Competing endogenous RNA (ceRNA) network construction

The predicted lncRNA‒miRNA pairs and miRNA‒mRNA pairs were collected from the miRcode and TargetScan databases. The lncRNA‒miRNA‒mRNA networks were visualized by Cytoscape. The predicted circRNA‒miRNA pairs were collected from the circbank database. The circRNA‒miRNA‒mRNA networks were visualized by Cytoscape. Furthermore, according to the lncRNA‒miRNA‒mRNA and circRNA‒miRNA‒mRNA networks, differentially expressed circRNAs, lncRNAs, and mRNAs that were regulated by the same miRNA were further screened to construct a ceRNA Network.

### Real-time quantitative PCR analysis

Two DEGs, four lncRNAs, four circRNAs and one miRNA from the ceRNA network were selected, and their differential expression was validated by real-time PCR. Total RNA was derived from human-luteinized granulosa (hGL) cells of PCOS and control. PCOS was diagnosed according to Rotterdam’s diagnostic criteria, which required the presence of any two of the following three conditions while excluding other causes of excessive androgen: 1) Oligo-ovulation or anovulation; 2) clinical manifestations of high androgen levels or hyperandrogenism; 3) polycystic ovaries identified by ultrasound, with one ovary or bilateral ovary showing a diameter of 2-9 mm follicles ≥ 12, or ovarian volume ≥ 10ml. The control group consisted of patients with tubal infertility. The reverse transcription of RNA was performed with the PrimeScript RT Reagent Kit (Perfect Real Time, Takara), following the manufacturer’s instructions. Then, qPCR was conducted to amplify cDNA samples using SYBR Green PCR Master Mix (#MR101; Vazyme, China; Q711; Vazyme, China). The expression levels of mRNAs, circRNAs and lncRNAs were normalized to GAPDH, and miRNA expression levels were normalized to U6 and analysed using the 2-ΔΔCt method. Sequences of the specific real-time PCR primers for selected genes and ncRNAs were presented in Supplemental Table 4.

### Cell culture

A GC tumor-derived cell line (KGN) was utilized to explore the miRNA‒mRNA network. KGN cells were seeded into six-well plates at a density of 2 × 10^5^ cells/well in DMEM supplemented with 5% foetal bovine serum. All the cell models used in this study were cultured in a humidified atmosphere of 5% CO2 and 95% air at 37 °C, and the cell culture medium was changed every 2 days in all experiments.

### Cell transfection

Cells were cultured to approximately 70% density and then transfected with 100 nM hsa-miR-205-5p mimics and negative control (RIBBIO, Guangzhou, China) using Lipofectamine RNA iMAX according to the manufacturer’s instructions (Life Technologies). The efficiency of the hsa-miR-205-5p mimics was detected by quantitative real-time RT‒PCR.

### Western blot analysis

After the treatment, all the cells were lysed in cell lysis buffer (Cell Signaling Technology), and the protein concentration was determined using a Pierce Rapid Gold BCA kit following the manufacturer’s instructions (Thermo Fisher, USA). Equal amounts of protein were loaded and separated using sodium dodecyl sulfate‒polyacrylamide gel electrophoresis (SDS‒PAGE) analysis. Afterwards, the proteins were transferred onto polyvinylidene difluoride (PVDF) membranes (Bio-Rad, USA), followed by blocking with Tris-buffered saline (TBS) containing 5% nonfat dry milk for 1 h at room temperature and incubated overnight at 4 °C with corresponding primary antibodies. The next day, the membranes were washed with TBS for 1 h and then incubated in the appropriate HRP-conjugated secondary antibody for 30 min. Similarly, the membranes were washed with TBS for 1 h after secondary antibody incubation. Finally, the immunoreactive bands were detected using an enhanced chemiluminescent substrate (Bio-Rad) and X-ray film. The intensities of the bands were quantified with Image-Pro Plus software (v.4.5; Media Cybernetics, USA).

### Statistical analysis

All experiments were repeated three times. Data analysis was performed using GraphPad Prism version 9 (GraphPad Software Inc, San Diego, CA, USA). All data are presented as the mean ± standard error of the mean (SEM). The Mann‒Whitney U test or unpaired Student’s t test was performed to compare mRNA, lncRNA, circRNA and miRNA expression between groups. Statistical significance was defined as a two-tailed *P* value < 0.05.

## Results

### Differential expression analysis and functional enrichment analysis

**Fig. 1:**
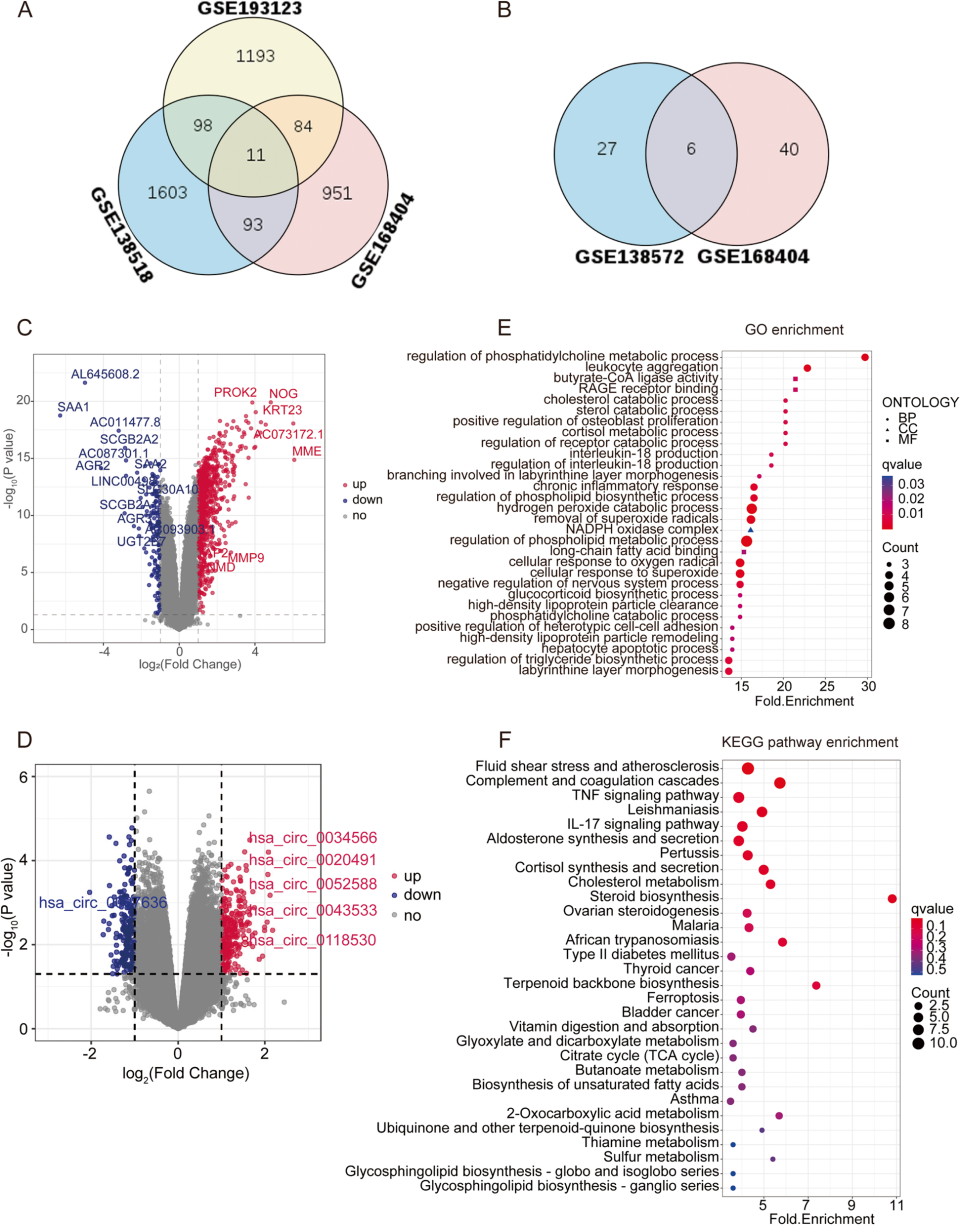
Differential Expression Analysis and Functional Enrichment Analysis. Venn diagram of three mRNA databases (**A**) and two miRNA databases (**B**). Volcano plot showing transcriptomic landscapes of lncRNAs (**C**) and circRNAs (**D**) in control and PCOS group. **E** Top 30 GO terms enriched by differentially expressed mRNAs. **F** Top 30 pathways enriched by differentially expressed mRNAs

According to the screening criteria, a total of 286 overlapping differentially expressed mRNAs (dif-mRNAs) in at least two mRNA datasets were obtained (Fig. 1A). Six overlapping differentially expressed miRNAs (dif-miRNAs) were identified (Fig. 1B). A total of 4256 differentially expressed lncRNAs (dif-lncRNAs) were obtained, of which 2634 were upregulated and 1622 were downregulated (Fig. 1C). A total of 3614 differentially expressed circRNAs (dif-circRNAs) were obtained, of which 1823 were upregulated and 1791 were downregulated (Fig. 1D). Then, functional enrichment analysis of dif-mRNAs was performed and the top 30 GO terms or KEGG pathways were displayed. We found that the GO terms that differentially expressed genes were mainly involved in were the regulation of phospholipid metabolic process, regulation of phosphatidylcholine metabolic process, and hydrogen peroxide catabolic process (Fig. 1E). Additionally, the KEGG pathways that the differentially expressed genes were significantly associated with included steroid biosynthesis, fluid shear stress and atherosclerosis, and complement and coagulation cascades (Fig. 1F).

### Protein-Protein Interaction (PPI) network, module extraction and hub genes identification

**Fig. 2:**
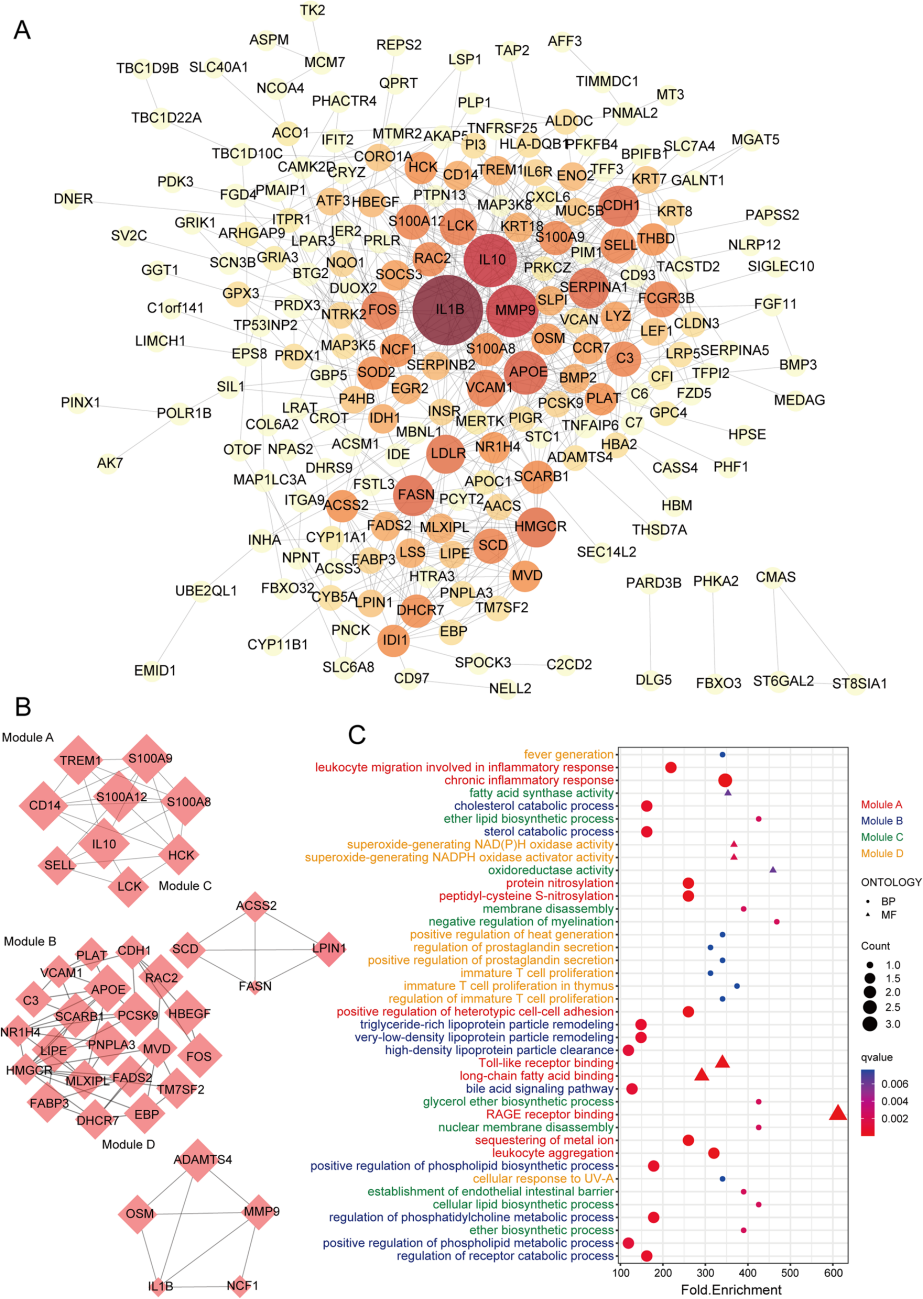
PPI network analysis of differentially expressed mRNAs and identification of hub mRNAs. **A** The PPI interaction network of DEGs that differed in PCOS. Each edge links two interacting genes. The size and the color depth of each node correlates positively with its degree of connectivity. **B** The top 4 significant modules were obtained from the PPI network. **C** Top 10 GO terms enriched by each modules gene

The PPI network based on dif-mRNA consisted of 202 nodes and 630 interaction pairs (Fig. 2A). Nodes with high topological scores can be regarded as key nodes of the network. Using the Cytoscape plug-in MCODE (score ≥ 4), four subnetwork modules were aggregated and extracted from the PPI network (Fig. 2B). Module A (score = 6) contained 9 nodes and 24 interaction pairs, in which inflammatory factors were most included, such as interleukin 10 (IL 10, degree = 70), LCK proto-oncogene, Src family tyrosine kinase (LCK, degree = 36), and CD14 molecule (CD14, degree = 20). Module B (score = 4.8) contained 21 nodes and 48 interaction pairs, in which the genes were associated with lipid metabolism, such as patatin-like phospholipase domain containing 3 (PNPLA3, degree = 12), mevalonate diphosphate decarboxylase (MVD, degree = 24), and fatty acid desaturase 2 (FADS2, degree = 20). Module C (score = 4) contained 4 nodes and 6 interaction pairs, including acyl-CoA synthetase short chain family member 2 (ACSS2, degree = 26) and stearoyl-CoA desaturase (SCD, degree = 32). Module D (score = 4) contained 5 nodes and 8 interaction pairs, including matrix metallopeptidase 9 (MMP9, degree = 68) and interleukin 1 beta (IL 1B, degree = 106). Moreover, genes in the modules were subjected to GO enrichment analysis. According to the significance order, the top 10 terms for each module were selected for display (Fig. 2C). The genes in Module A were significantly involved in the chronic inflammatory response, Toll-like receptor binding, and RAGE receptor binding. Genes in Module B were significantly associated with cholesterol catabolic process and sterol catabolic process. Genes in Module C were concerned with oxidoreductase activity and cellular lipid biosynthetic process. Genes in Module D were enriched in superoxide-generating NAD(P)H oxidase activity and immature T-cell proliferation in thymus.

**Fig. 3:**
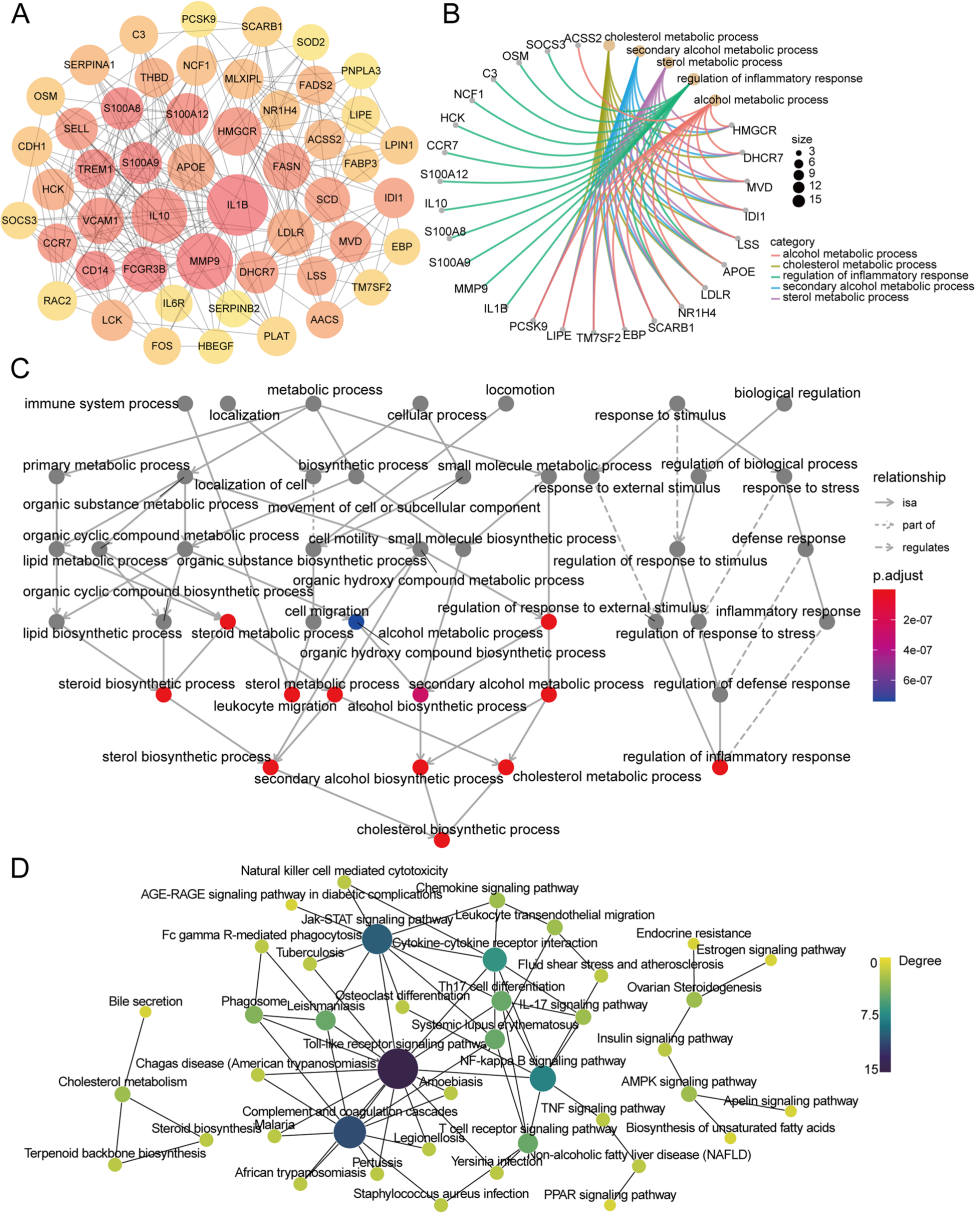
Fnctional enrichment analysis of hub mRNAs. **A** Top 50 hub genes ranked by MCC from PPI network. **B** Top 5 GO-BP terms enriched by 50 hub genes. **C** GO-Tree network analysis based on the interaction relationship of enriched BP terms. **D** Pathway-act network analysis illustrated mutual interactions between pathway terms

To further identify the hub genes, we identified hub mRNAs by cytoHubba, a plug in Cytoscape, and the top 50 hub genes were identified and selected from the PPI network (Fig. 3A). GO enrichment analysis showed that hub mRNAs were mainly enriched in cholesterol metabolic process, sterol metabolic process, and regulation of inflammatory response (Fig. 3B). GO-Tree analysis of GO biological process (GO-BP) terms was performed to further understand the core BPs associated with PCOS based on their subordinate and interaction relationships (Fig. 3C). A Pathway-Act network was constructed to further investigate the mutual interactions of pathways and to obtain the hub pathways that may play a vital role in PCOS (Fig. 3D). The top pathways that showed interactions with other surrounding pathways were the Toll-like receptor signaling pathway, cholesterol metabolism, ovarian steroidogenesis, and the AMPK signaling pathway. These results indicated that lipid metabolism, especially the cholesterol metabolic pathway, and regulation of the inflammatory response, especially the Toll-like receptor signaling pathway, might play key roles in PCOS initiation and progression.

### ceRNA network construction

**Fig. 4:**
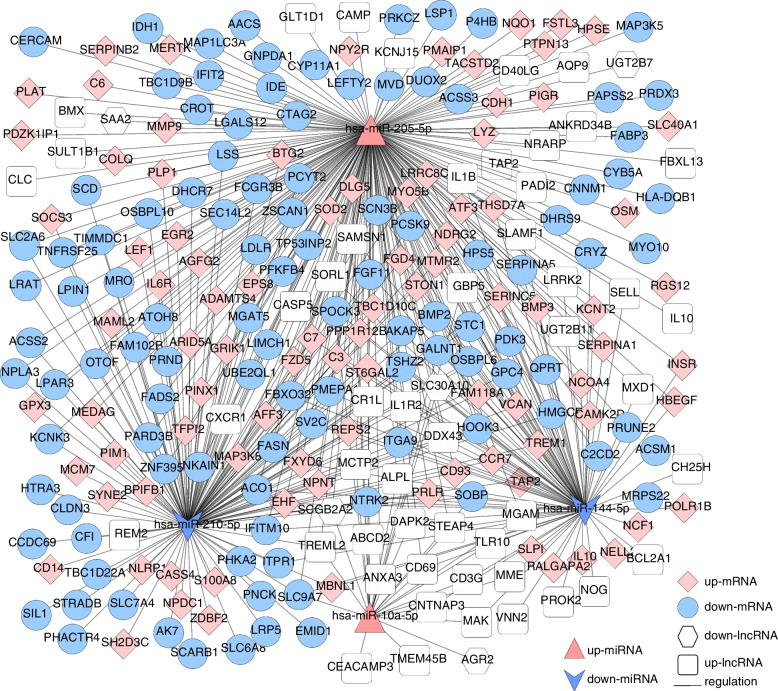
The lncRNA–miRNA–mRNA Network. Pink prism represents the upregulated mRNAs, blue circle indicates downregulated genes, red triangle indicates upregulated miRNAs, blue arrow shows the downregulated miRNAs, and white quadrilateral indicates the upregulated lncRNAs, white hexagon indicates the downregulated lncRNAs

Based on the regulatory relationship of dif-miRNA–dif-mRNA and dif-miRNA–dif-lncRNA, the lncRNAs and mRNAs that were significantly differentially expressed and regulated by the same miRNA were screened. In total, 457 lncRNA–miRNA–mRNA interactions were finally obtained (Fig. 4 and Supplement Table 1), including 50 upregulated and 5 downregulated lncRNAs, 91 upregulated and 110 downregulated mRNAs, and 2 upregulated and 2 downregulated miRNAs.

**Fig. 5:**
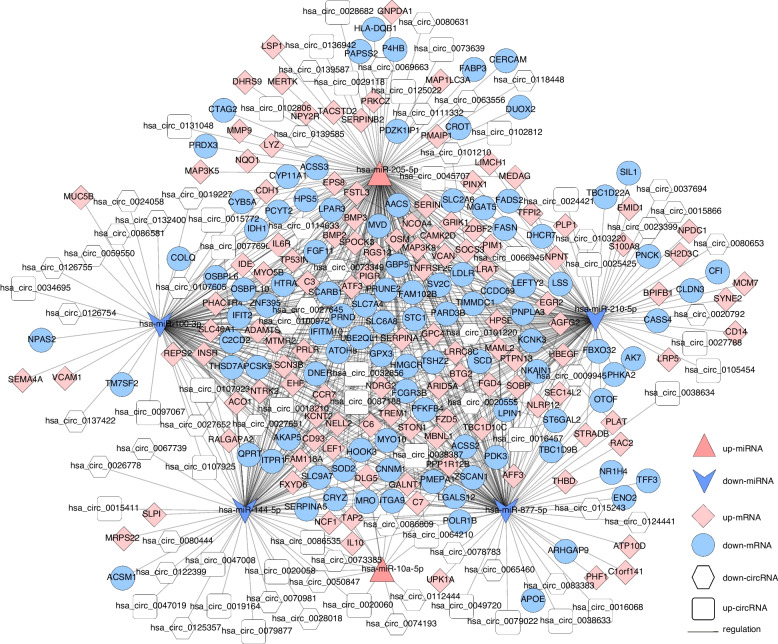
The circRNA–miRNA–mRNA Network. Pink prism represents the upregulated mRNAs, blue circle indicates downregulated genes, red triangle indicates upregulated miRNAs, blue arrow shows the downregulated miRNAs, and white quadrilateral indicates the upregulated circRNAs, white hexagon indicates the downregulated circRNAs

Based on the regulatory relationship of dif-miRNA–dif-mRNA and dif-miRNA–dif-circRNA, dif-circRNA and mRNA regulated by the same miRNAs were screened, resulting in 707 interaction relationships of circRNA–miRNA–mRNA. There were 39 upregulated and 49 downregulated circRNAs, 117 upregulated mRNAs and 101 downregulated mRNAs, and 2 upregulated miRNAs and 4 downregulated miRNAs. The circRNA–miRNA–mRNA network is shown in Fig. 5 and Supplemental Table 2.

**Fig. 6:**
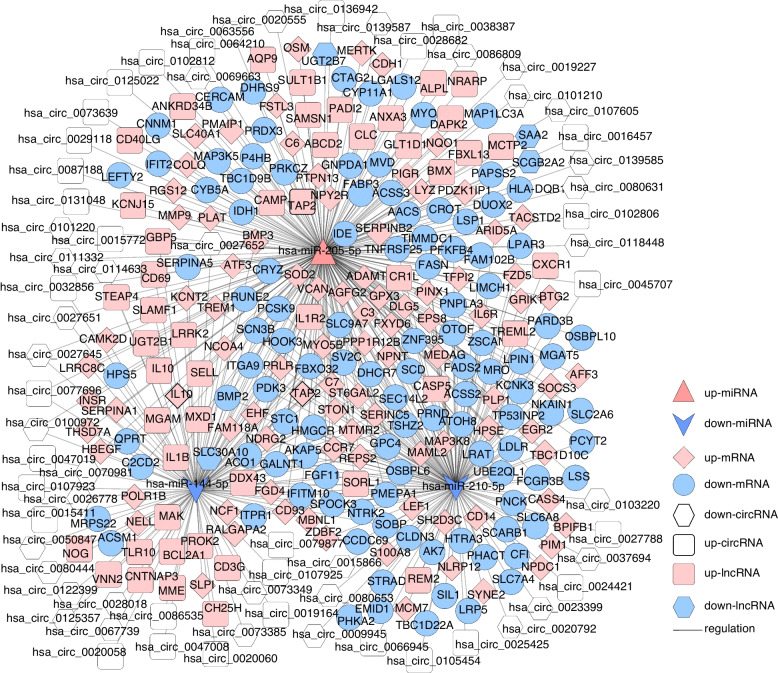
The Competing Endogenous RNA (ceRNA) Network. Pink prism represents the upregulated mRNAs, blue circle indicates downregulated genes, red triangle indicates upregulated miRNAs, blue arrow shows the downregulated miRNAs, white quadrilateral indicates the upregulated circRNAs, white hexagon indicates the downregulated circRNAs, and pink quadrilateral indicates the upregulated lncRNAs, blue hexagon indicates the downregulated lncRNAs

Furthermore, according to the lncRNA–miRNA–mRNA and circRNA–miRNA–mRNA networks, differentially expressed circRNAs, lncRNAs, and mRNAs that were regulated by the same miRNA were further screened. Finally, 504 interaction pairs were obtained (Fig. 6 and Supplement Table 3), of which 31 upregulated and 35 downregulated circRNAs, 48 upregulated and 4 downregulated lncRNAs, 91 upregulated and 109 downregulated mRNAs, and 1 upregulated and 2 downregulated miRNAs (hsa-miR-144-5p, and hsa-miR-210-5p, downregulated; hsa-miR-205-5p, upregulated) were included.

### RT–qPCR validation

**Fig. 7:**
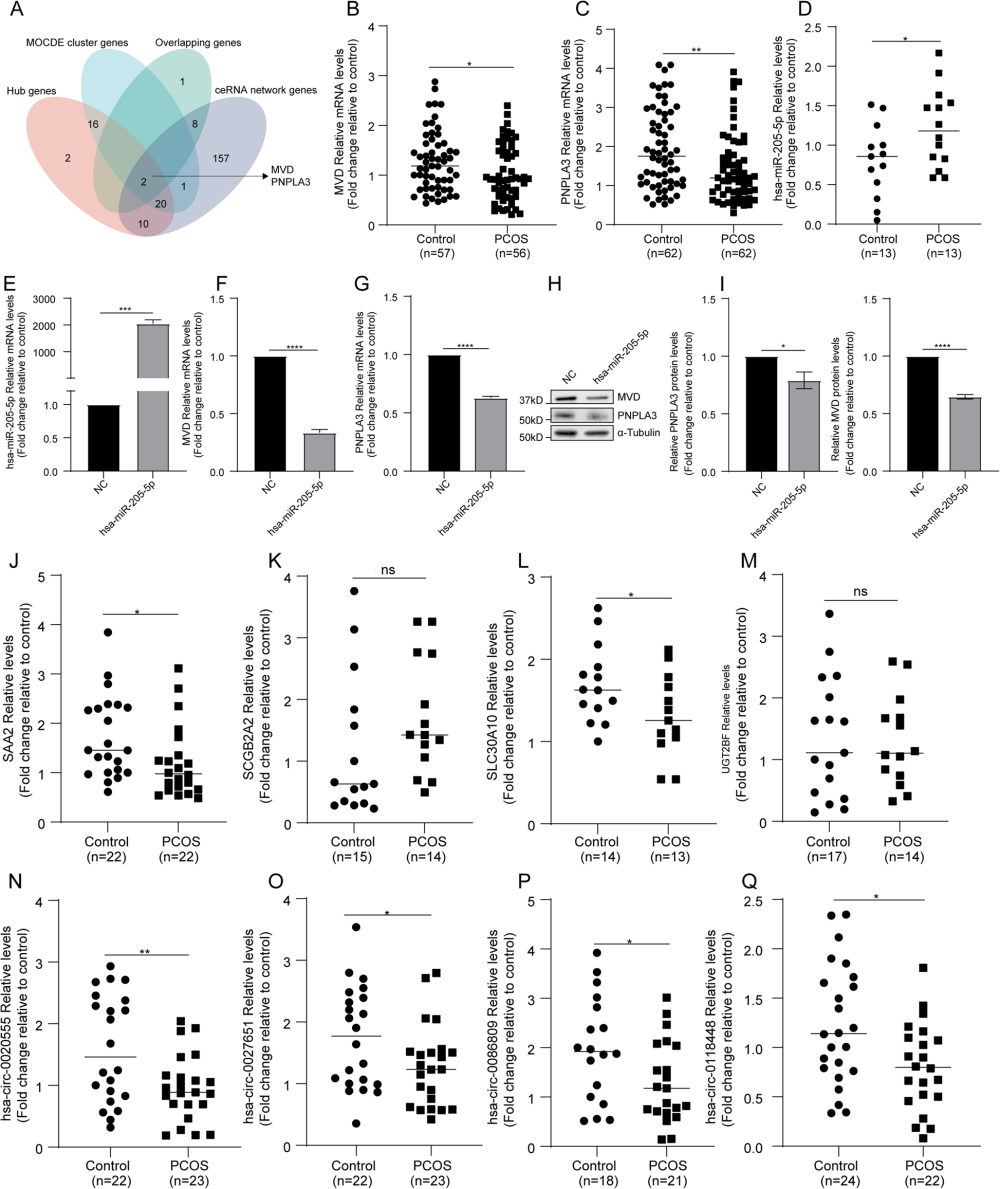
The expression of DEGs and differentially expressed ncRNAs in hGL and KGN. **A** Venn diagram of Hub genes, MCODE cluster genes, 11 overlapping genes of three mRNA databases, and mRNAs in ceRNA network. The mRNA expression levels of MVD (*n* = 113) (**B**), PNPLA3 (*n* = 124) (**C**), hsa-miR-205-5p (*n* = 26) in hGL (**D**). KGN were transfected with hsa-miR-205-5p mimics (100nM), and the expression of MVD and PNPLA3 were examined by RT–PCR and western blot respectively (**E**-**I**). The mRNA expression levels of SAA2 (*n* = 44) (**J**), SCGB2A2 (*n* = 29) (**K**), SLC30A10 (*n* = 27) (**L**), UGT2BF (*n* = 31) (**M**), hsa-circ-0020555 (*n* = 45) (**N**), hsa-circ-0027651 (*n* = 45) (**O**), hsa-circ-0086809 (*n* = 39) (**P**), hsa-circ-0118448 (*n* = 46) in hGL (**Q**)

To further narrow down the hub dif-mRNAs to identify those that are the core dif-mRNAs, we merged the hub dif-mRNAs from the PPI network, MCODE cluster mRNAs, 11 overlapping mRNAs, and dif-mRNAs in the ceRNA network and found two core dif-mRNAs (Fig. 7A). These core mRNAs were all downregulated in PCOS and regulated by hsa-miR-205-5p, including the triacylglycerol lipase PNPLA3 and cholesterol biosynthesis enzyme MVD. To fully validate these most differentiated mRNAs, lncRNAs, miRNAs and circRNAs, we performed RT–qPCR using hGL cells from Women’s Hospital, School of Medicine, Zhejiang University. The primers for real-time quantitative PCR are shown in Supplemental Table 4. The baseline characteristics of the patients are shown in Table 2. AFC, BMI, duration of infertility, LH level, ratio of LH to FSH, T level, AMH, triglyceride, Total cholesterol, LDL-C, HOMA-IR, number of oocytes retrieved, and number of MII oocytes were higher in the PCOS group than in the control group. The number of 2PN fertilized oocytes was lower in the PCOS group. We observed downregulation of PNPLA3 and MVD (Fig. 7B and C), and upregulation of hsa-miR-205-5p in hGL (Fig. 7D). Moreover, transfection of KGN cells with hsa-miR-205-5p mimics led to downregulation of PNPLA3 and MVD at both mRNA and protein levels, supporting the regulatory relationship between hsa-miR-205-5p and PNPLA3 and MVD (Fig. 7E-I). Additionally, we found that two lncRNAs (SAA2 and SLC30A10) and four circRNAs (hsa-circ-0020555, hsa-circ-0027651, hsa-circ-0086809, and hsa-circ-0118448) which regulate hsa-miR-205-5p were all downregulated in hGL (Fig. 7J-M, N-Q).

**Table 2:** Baseline characteristics of the participants

Characteristic	Control (*n* = 66)	PCOS (*n* = 64)	*P* value
Age (years)	30.45 ± 3.71	30.03 ± 3.68	0.515
BMI (kg/m2)	21.76 ± 3.10	22.87 ± 3.10	**0.044**
Duration of infertility (years)	2.57 ± 2.14	3.83 ± 2.51	**0.004**
Antral follicle count	13.31 ± 4.88	19.70 ± 6.86	**0.000**
Laboratory tests			
Ratio of LH to FSH	0.98 ± 0.76	1.47 ± 0.92	**0.001**
LH (IU/L)	6.39 ± 6.71	8.63 ± 5.14	**0.036**
FSH (IU/L)	6.27 ± 1.45	6.07 ± 1.71	0.472
Estradiol (pmol/L)	88.40 ± 47.65	92.51 ± 51.42	0.640
TT (nmol/L)	0.69 ± 0.34	1.34 ± 0.66	**0.000**
P (nmol/L)	1.41 ± 1.46	1.11 ± 1.88	0.310
PRL (ng/ml)	24.25 ± 8.56	19.54 ± 17.05	0.165
AMH (ng/mL)	4.36 ± 2.18	8.37 ± 4.82	**0.000**
Triglyceride (mmol/ L)	1.05 ± 0.47	1.54 ± 1.09	**0.001**
Total cholesterol (mmol/L)	4.11 ± 0.61	4.92 ± 2.16	**0.004**
HDL-C (mmol/L)	1.41 ± 0.25	1.32 ± 0.30	0.071
LDL-C (mmol/L)	2.37 ± 0.48	2.58 ± 0.61	**0.028**
Fasting glucose (mmol/L)	4.61 ± 0.75	4.87 ± 0.57	0.108
Fasting insulin (µU/ mL)	7.33 ± 3.49	9.98 ± 6.78	0.430
HOMA-IR	1.61 ± 0.65	2.31 ± 1.44	**0.001**
Duration of gonadotropin stimulation (d)	9.91 ± 1.73	11.23 ± 2.61	**0.001**
Total dose of gonadotropin (IU)	2077.27 ± 664.73	2085.12 ± 813.93	0.952
Type of fertilization— no. (%)			0.923
With IVF	33 (50.00%)	32 (50.00%)	
With ICSI	23 (34.85%)	23 (35.94%)	
With mixed IVF and ICSI	10 (15.15%)	9 (14.06%)	
No. of oocytes retrieved	13.32 ± 5.63	17.78 ± 9.21	**0.001**
No. of MII oocytes	10.97 ± 5.29	15.05 ± 8.49	**0.001**
Rate of MII oocytes	0.84 ± 0.21	0.86 ± 0.19	0.712
No. of 2 PN fertilized	7.91 ± 4.71	9.58 ± 6.47	0.095
2PN fertilization rate	0.72 ± 0.22	0.62 ± 0.25	**0.015**
No. of day 3 good-quality embryos	3.98 ± 2.23	4.47 ± 2.94	0.292
Rate of day 3 good-quality embryos	0.57 ± 0.25	0.52 ± 0.27	0.236

Data are presented as the mean ± standard deviation, or number (percentage) of cases

*BMI* Body mass index, *LH* Luteinizing hormone, *FSH* Follicle-stimulating hormone, *TT* Total testosterone, *AMH* Anti-mullerian hormone, *P* Progesterone, *PRL* Prolactin, *HDL* High-density lipoprotein, *LDL* Low-density lipoprotein, *HOMA-IR* Homeostatic model assessment of insulin resistance, *IVF* In vitro fertilization, *ICSI* Intracytoplasmic sperm injection, *MII* Mature oocytes, *2PN* fertilized oocytes with two primary pronucleus

*P*-value from analysis of variance or independent t-test for continuous variables, and Chi-square test or Fisher’s exact test for categorical variables. *p* < 0.05 was considered statistically significant

**Fig. 8:**
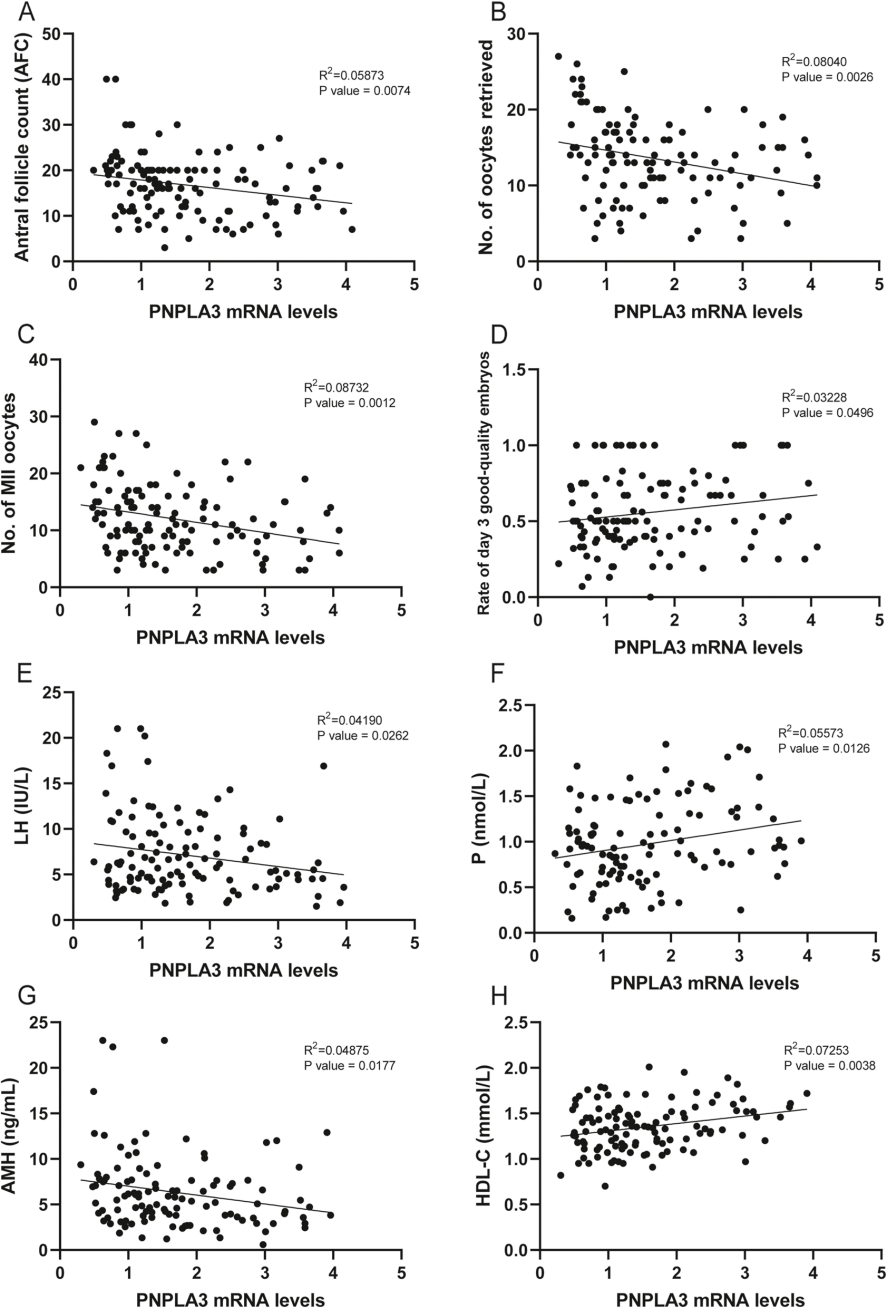
The PNPLA3 mRNA levels with patients’ clinical characteristics. The correlation analysis of PNPLA3 mRNA levels with AFC (**A**), No. of oocytes retrieved (**B**), No. of MII oocytes (**C**), Rate of day 3 good-quality embryos (**D**), LH levels (**E**), P levels (**F**) AMH levels (**G**), HDL-C (**H**)

**Fig. 9:**
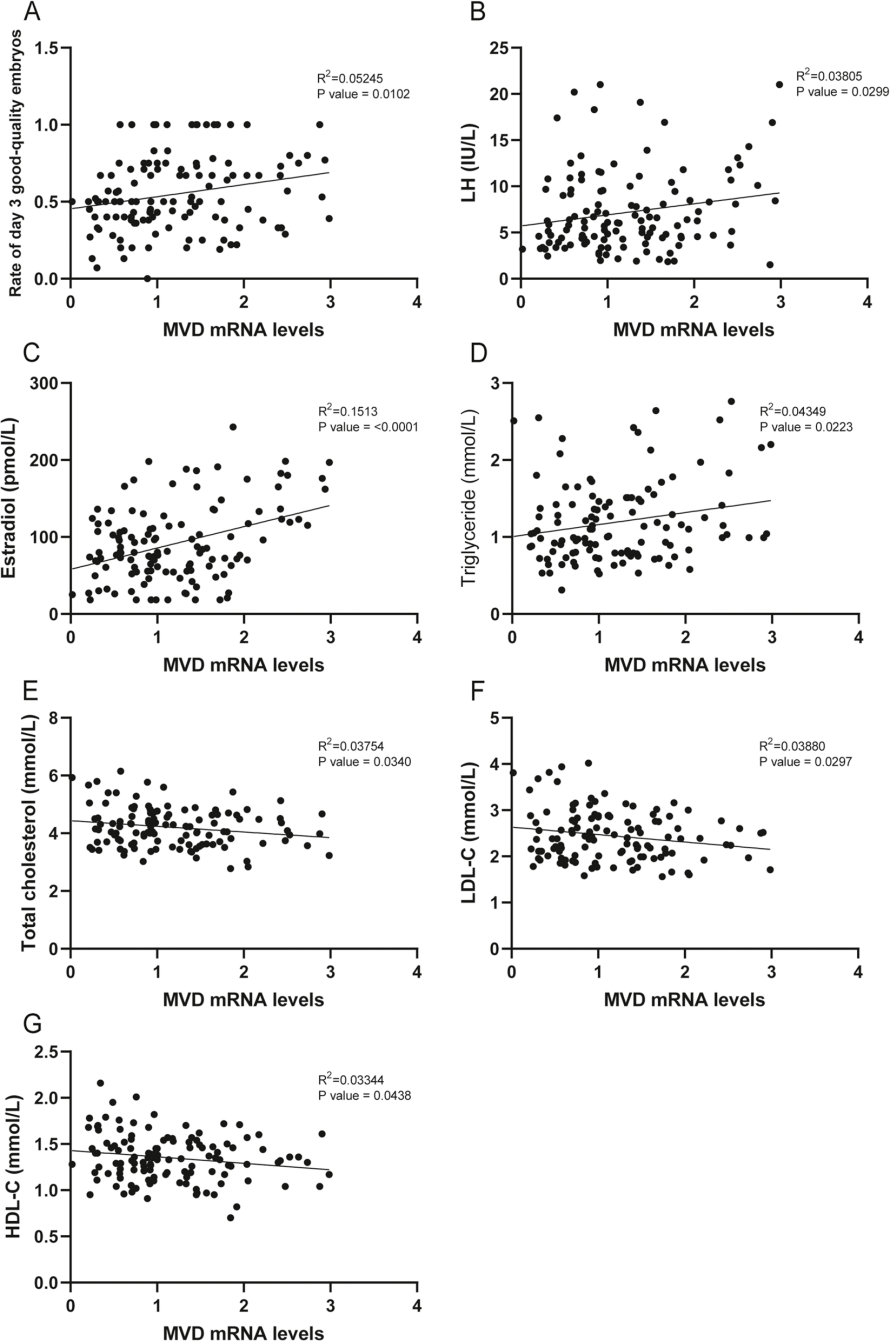
The MVD mRNA levels with patients’ clinical characteristics. The correlation analysis of MVD mRNA levels with Rate of day 3 good-quality embryos (**A**), LH levels (**B**), Estradiol (**C**), Triglyceride (**D**), Total cholesterol (**E**), LDL-C (**F**), HDL-c (**G**)

To further explore the relationship between PNPLA3 and MVD and the occurrence and progression of PCOS, we correlated the mRNA expression of these two genes with the clinical characteristics of patients (Table 3). We found that PNPLA3 was negatively correlated with AFC, number of oocytes retrieved, number of MII oocytes, LH levels, and AMH levels, and positively correlated with the rate of day 3 good-quality embryos, P levels and HDL-C levels (Fig. 8A-H, Figure S1). MVD was negatively correlated with total cholesterol levels, LDL-C levels and HDL-C levels, and positively correlated with the rate of day 3 good-quality embryos, LH levels, estradiol levels and triglyceride levels (Fig. 9A-H, Figure S2). MVD is a key enzyme in cholesterol synthesis, and cholesterol is an important precursor for steroid hormone synthesis, hence, we speculate that reduced MVD levels in PCOS affect cholesterol synthesis and further affect the synthesis of steroid hormones such as estrogen, which in turn affects oocyte development, but the mechanism still needs to be further explored.

**Table 3:** The Pearson correlation analysis of PNPLA3 and MVD with clinical characteristics

Characteristic	PNPLA3		MVD	
	R^2^	*P* value	R^2^	*P* value
BMI (kg/m^2^)	0.02719	0.0707	0.00002	0.9608
Antral follicle count	0.05873	**0.0074**	0.00190	0.6292
No. of oocytes retrieved	0.08040	**0.0026**	0.01286	0.2043
No. of MII oocytes	0.08732	**0.0012**	0.00081	0.7495
No. of 2 PN fertilized	0.05045	**0.0145**	0.00145	0.6707
No. of day 3 good-quality embryos	0.00891	0.2971	0.01622	0.1536
Rate of MII oocytes	0.09386	**0.0272**	0.00729	0.5556
2PN fertilization rate	0.00214	0.6282	0.07854	0.7798
Rate of day 3 good-quality embryos	0.04943	**0.0233**	0.05245	**0.0102**
LH (IU/L)	0.04190	**0.0262**	0.03805	**0.0299**
FSH (IU/L)	0.00080	0.7594	0.00223	0.6026
Estradiol (pmol/L)	0.00220	0.6127	0.1513	**< 0.0001**
P (nmol/L)	0.05573	**0.0126**	0.00051	0.8080
TT (nmol/L)	0.05747	0.0779	0.00202	0.7377
AMH (ng/mL)	0.04875	**0.0177**	0.00098	0.7391
Triglyceride (mmol/L)	0.00420	0.4931	0.04349	**0.0223**
Total cholesterol (mmol/L)	0.00785	0.3575	0.03754	**0.0340**
HDL-C (mmol/L)	0.07253	**0.0038**	0.03344	**0.0438**
LDL-C (mmol/L)	0.00440	0.4793	0.03880	**0.0297**
HOMA-IR	0.01033	0.3071	0.00511	0.4622

The correlation analysis of PNPLA3 and MVD with clinical characteristics

*BMI* Body mass index, *LH* Luteinizing hormone, *FSH* Follicle-stimulating hormone, *TT* Total testosterone, *AMH* Anti-mullerian hormone, *P* Progesterone, *HDL* High-density lipoprotein, *LDL* Low-density lipoprotein, *HOMA-IR* Homeostatic model assessment of insulin resistance, *MII* Mature oocytes, *2PN* fertilized oocytes with two primary pronucleus

*p* < 0.05 was considered statistically significant

## Discussion

The current study aimed to identify common DEGs of PCOS from multiple datasets and to identify the potential genes responsible for the occurrence and progression of PCOS. In the present study, we analysed the whole-transcriptome characteristics of PCOS GCs. The GO and KEGG pathway analyses showed that cholesterol catabolic process, sterol catabolic process, and chronic inflammatory response were significantly enriched. To further explore the interactions between mRNA and ncRNA during the pathogenesis of PCOS, we constructed PPI and ceRNA networks. It turned out that mRNA (PNPLA3 and MVD), miRNA (hsa-miR-205-5p, hsa-miR-210-5p, has-and miR-144-5p), and miRNA-associated lncRNAs and circRNAs play key roles in the initiation and progression of PCOS. Overall, we delineated the GCs whole-transcriptomic landscape and identified possible changes in ovarian steroidogenesis, metabolic disorders, and immune dysfunction in PCOS.

The cross-talk between GCs and follicles plays a key role in follicular development and maturation [25]. PCOS patients show blocked follicle development in the small sinus follicular phase and polycystic ovaries [26]. In the early stage of follicle development, follicles are mainly composed of immature oocytes and GCs surrounding them [27]. Studies have shown that in the process of primordial follicle activation and follicle development, the metabolism of oocytes and surrounding GCs is significantly enhanced [28, 29]. Metabolism disorders such as lipid metabolism disorders or hyperandrogenemia can cause follicular development arrest [30]. In the current study, we found that metabolic processes and ovarian steroidogenesis were significantly impaired. Cholesterol catabolic processes and sterol catabolic processes were significantly enriched in PCOS GCs. Among the genes we identified through coexpression of three datasets, PNPLA3, MVD, MMP9, oncostatin M (OSM), LCK, triggering receptor expressed on myeloid cells 1 (TREM1), FADS2, proprotein convertase subtilisin/kexin type 9 (PCSK9), and C3 are involved in lipid metabolism and ovarian dysfunction. FADS2 is decreased in the transcriptome sequencing of PCOS patients with androgen excess and regulates lipid metabolism of PCOS patients [14, 31]. In addition, PNPLA3 may regulate oocyte development capacity in PCOS [32]. Abnormal high expression of proprotein PCSK9 may be involved in pathogenesis of PCOS by affecting lipid metabolism and ovarian function, and the inhibition of PCSK9 partly reverses the pathological changes in PCOS [33–35]. MMP9 and TREM1 were identified as hub genes in PCOS patients with non-alcoholic fatty liver (NAFLD). Upregulation of MMP9 could contribute to excess abdominal adiposity and hyperandrogenism, which might be related to increased cardiovascular risk in PCOS [36–38]. Metformin could decrease MMP9 levels to improve the lipid metabolism of PCOS [39, 40]. OSM, a recently identified adipokine, could negatively affect oocyte maturation and fertilization rates [41]. The tyrosine kinase LCK may have an effect on PCOS patients with insulin resistance (IR) and may play an important role in PCOS pathogenesis [42]. On all accounts, our results showed that abnormal lipid metabolism was closely related to the occurrence and development of PCOS; however, the specific mechanism underlying how these metabolic disorders contribute to ovulatory disorders and follicular atresia in PCOS remains elusive. These genes involved in metabolic disorders in PCOS GCs modulated cholesterol synthesis and ovarian steroidogenesis, which indicated that fatty acid and cholesterol metabolism disorders in PCOS GCs may contribute to the impairment in oocyte maturation.

In recent years, several studies have shown that women with PCOS present with chronic low-grade inflammation, indicating abnormal expression of the proinflammatory cytokines interleukin-1 (IL-1), CRP, and interleukin-18 (IL-18), as well as endothelial dysfunction and increased oxidative stress [43–46]. Anti-inflammatory therapy can improve the symptoms of PCOS [47, 48]. We found that the chronic inflammatory response was enriched and that IL-18 production was increased, which indicated an immune imbalance in PCOS patients. In addition, C3 was involved in the regulation of inflammation. Higher C3 levels also had a stronger association with IR and might be an inflammatory marker of IR in women with PCOS [49, 50]. Women with PCOS exhibited higher MMP9 levels, which could contribute to chronic low-grade inflammation and an atherothrombotic state [37]. Moreover, in PCOS offspring, MMP9 was also increased, suggesting that these children may exhibit increased chronic low-grade inflammation [51]. In the physiological state, GCs excrete prostaglandin E2 (PGE2) and some inflammatory cytokines and chemokines to promote ovulation [52]. These genes are involved in immune regulation to maintain the immune balance of GCs in PCOS patients, which could support follicle development and ovulation. The dysregulation of genes associated with immune regulation could be a molecular clue regarding the abnormal folliculogenesis and anovulation in PCOS patients.

The circRNA dataset (GSE145296) was used cumulus cells (CCs), while the others were used mural granulosa cells (MGCs). This dataset is currently the only one in the GEO database performing circRNA sequencing on granulosa cells from PCOS patients. CircRNAs play an essential role in whole transcriptome analysis and have significant physiological functions, such as regulating mRNA expression by acting as competitive endogenous RNAs for miRNAs, which can impact cellular function and contribute to PCOS development [21, 22, 53, 54]. In addition, undifferentiated GCs differentiate into MGCs and CCs during follicle antrum formation based on their location distribution [55]. The oocyte within the follicle is surrounded by CCs, which connect with MGCs that are responsible for secreting steroid hormones. Crosstalk between oocytes and surrounding somatic cells is crucial for mammalian oogenesis [25, 56]. Both cell types originate from undifferentiated GCs, the transcriptomes of these cell types differ before ovulation [57, 58], but they converge after ovulation such that their gene expression profiles become completely overlapping. Notably, CCs transition to be more like MGCs after human chorionic gonadotropin (HCG) [59]. The CCs used in the circRNA dataset (GSE145296) was retrieved 36 h after HCG administration. Hence, the CCs and MGCs could have similar gene expression profiles. Thus, we kept this dataset for integrated analysis. We also validated the differential circRNAs identified in the dataset using hGL. The results showed that all four circRNAs were downregulated. Thus, using this dataset of CCs for complete transcriptome analysis provides valuable insights into changes in the whole transcriptome of GCs in PCOS.

## Conclusion

In summary, this study found several DEGs related to metabolic processes, ovarian steroidogenesis and the immune response, including MVD, PNPLA3, MMP9, OSM, and C3. Additionally, this study identified some ncRNAs, especially hsa-miR-205-5p and its associated circRNAs and lncRNAs. The relationship among these molecules may contribute to the onset and development of PCOS. We delineated the GC whole-transcriptomic landscape and provided a valuable direction aiming to improve the fertility of PCOS in China.

### Supplementary Information

**Additional file 1: Supplemental Table 1.** The lncRNA-miRNA-mRNA Network.

**Additional file 2: Supplemental Table 2.** The circRNA-miRNA-mRNA Network.

**Additional file 3: Supplemental Table 3.** The Competing Endogenous RNA (ceRNA) Network.

**Additional file 4: Supplemental Table 4.** The primers for real-time quantitative PCR.

**Additional file 5: Supplemental Table 5.** Antibody information.

**Additional file 6: Supplemental Figure 1.** The PNPLA3 mRNA levels with patients’ clinical characteristics. The correlation analysis of PNPLA3 mRNA levels with BMI (A), No. of 2PN fertilized (B), No. of day 3 good-quality embryos (C), 2PN fertilization rate (D), FSH levels (E), Estradiol (F) TT levels (G), Triglyceride (H), Total cholesterol (I), LDL-C (J), HOMA-IR (K).

**Additional file 7: Supplemental Figure 2.** The MVD mRNA levels with patients’ clinical characteristics. The correlation analysis of MVD mRNA levels with BMI (A), AFC (B), No. of oocytes retrieved (C), No. of MII oocytes (D), No. of 2PN fertilized (E), No. of day 3 good-quality embryos (F), 2PN fertilization rate (G), FSH levels (H), P levels (I), TT levels (J), AMH levels (K), HOMA-IR (L).

## Data Availability

The microarray datasets GSE138518, GSE168404, GSE193123, GSE138572, GSE95728, GSE145296 were downloaded from GEO database (https://www.ncbi.nlm.nih.gov/geo/).

## Abbreviations

PCOS: Polycystic ovary syndrome
LH: Luteinizing hormone
IVF: In vitro fertilization
ICSI: Intracytoplasmic sperm injection
MII: Mature oocytes
2PN: Fertilized oocytes with two primary pronucleus
GN: Gonadotropin
FSH: Follicle stimulating hormone
AFC: Antral follicle count
BMI: Body mass index
E2: Estradiol
P: Progestin
PRL: Prolactin
HDL: High-density lipoprotein
LDL: Low-density lipoprotein
HOMA-IR: Homeostatic model assessment of insulin resistance
HCG: Human chorionic gonadotropin
CC: Cumulus cell
MGC: Mural granulosa cells
