# Supplementary material for: Integrated bioinformatics analysis elucidates granulosa cell whole-transcriptome landscape of PCOS in China
Source: J Ovarian Res. 2023 Aug 3;16:154. doi: 10.1186/s13048-023-01223-0 (PMC10398987; doi:10.1186/s13048-023-01223-0)
Supplement: Supplementary file 1 — Additional file 1: Supplemental Table 1. The lncRNA-miRNA-mRNA Network. [file 13048_2023_1223_MOESM1_ESM.pdf]

The lncRNA-miRNA-mRNA Network

miRNA	name
hsa-miR-205-5p	SLAMF1
hsa-miR-205-5p	ANXA3
hsa-miR-205-5p	CD69
hsa-miR-205-5p	IL10
hsa-miR-205-5p	FBXL13
hsa-miR-205-5p	CXCR1
hsa-miR-205-5p	LRRK2
hsa-miR-205-5p	SCGB2A2
hsa-miR-205-5p	CLC
hsa-miR-205-5p	KCNJ15
hsa-miR-205-5p	SAMSN1
hsa-miR-205-5p	SLC30A10
hsa-miR-205-5p	TREML2
hsa-miR-205-5p	ABCD2
hsa-miR-205-5p	MGAM
hsa-miR-205-5p	NRARP
hsa-miR-205-5p	DAPK2
hsa-miR-205-5p	MXD1
hsa-miR-205-5p	STEAP4
hsa-miR-205-5p	MCTP2
hsa-miR-205-5p	GLT1D1
hsa-miR-205-5p	ALPL
hsa-miR-205-5p	SORL1
hsa-miR-205-5p	CR1L
hsa-miR-205-5p	BMX
hsa-miR-205-5p	IL1R2
hsa-miR-205-5p	SELL
hsa-miR-205-5p	SULT1B1
hsa-miR-205-5p	ANKRD34B
hsa-miR-205-5p	CAMP
hsa-miR-205-5p	UGT2B7
hsa-miR-205-5p	SAA2
hsa-miR-205-5p	TAP2
hsa-miR-205-5p	DDX43
hsa-miR-205-5p	AQP9
hsa-miR-205-5p	PADI2
hsa-miR-205-5p	CD40LG
hsa-miR-205-5p	IL1B
hsa-miR-205-5p	CASP5
hsa-miR-205-5p	UGT2B11
hsa-miR-144-5p	MAK
hsa-miR-144-5p	MGAM
hsa-miR-144-5p	IL1R2
hsa-miR-210-5p	CR1L
hsa-miR-210-5p	SORL1
hsa-miR-144-5p	DDX43
hsa-miR-210-5p	REM2
hsa-miR-144-5p	GBP5
hsa-miR-144-5p	MXD1
hsa-miR-144-5p	CH25H
hsa-miR-144-5p	BCL2A1
hsa-miR-144-5p	SELL
hsa-miR-144-5p	CD3G
hsa-miR-144-5p	NOG
hsa-miR-144-5p	LRRK2
hsa-miR-144-5p	CD69

hsa-miR-144-5p	STEAP4
hsa-miR-144-5p	CNTNAP3
hsa-miR-144-5p	SLC30A10
hsa-miR-210-5p	TREML2
hsa-miR-144-5p	TLR10
hsa-miR-144-5p	SORL1
hsa-miR-144-5p	IL1B
hsa-miR-144-5p	VNN2
hsa-miR-210-5p	CASP5
hsa-miR-144-5p	PROK2
hsa-miR-144-5p	MME
hsa-miR-144-5p	SLAMF1
hsa-miR-210-5p	CXCR1
hsa-miR-210-5p	DDX43
hsa-miR-144-5p	UGT2B11
hsa-miR-210-5p	IL1R2
hsa-miR-10a-5p	SAMSN1
hsa-miR-10a-5p	SCGB2A2
hsa-miR-10a-5p	CD69
hsa-miR-10a-5p	MGAM
hsa-miR-10a-5p	SLC30A10
hsa-miR-10a-5p	AGR2
hsa-miR-10a-5p	GBP5
hsa-miR-10a-5p	MME
hsa-miR-10a-5p	TREML2
hsa-miR-10a-5p	TMEM45B
hsa-miR-10a-5p	MAK
hsa-miR-10a-5p	DAPK2
hsa-miR-10a-5p	ABCD2
hsa-miR-10a-5p	STEAP4
hsa-miR-10a-5p	CR1L
hsa-miR-10a-5p	SORL1
hsa-miR-10a-5p	MCTP2
hsa-miR-10a-5p	CEACAMP3
hsa-miR-10a-5p	CD3G
hsa-miR-10a-5p	TLR10
hsa-miR-10a-5p	CNTNAP3
hsa-miR-10a-5p	ALPL
hsa-miR-10a-5p	ANXA3
hsa-miR-205-5p	AACS
hsa-miR-205-5p	ACO1
hsa-miR-144-5p	ACO1
hsa-miR-210-5p	ACO1
hsa-miR-144-5p	ACSM1
hsa-miR-205-5p	ACSS2
hsa-miR-210-5p	ACSS2
hsa-miR-205-5p	ACSS3
hsa-miR-205-5p	ADAMTS4
hsa-miR-210-5p	ADAMTS4
hsa-miR-205-5p	AFF3
hsa-miR-210-5p	AFF3
hsa-miR-205-5p	AGFG2
hsa-miR-210-5p	AGFG2
hsa-miR-210-5p	AK7
hsa-miR-205-5p	AKAP5
hsa-miR-144-5p	AKAP5
hsa-miR-210-5p	AKAP5
hsa-miR-205-5p	ARID5A

hsa-miR-210-5p	ARID5A
hsa-miR-205-5p	ATF3
hsa-miR-144-5p	ATF3
hsa-miR-205-5p	ATOH8
hsa-miR-210-5p	ATOH8
hsa-miR-205-5p	BMP2
hsa-miR-210-5p	BMP2
hsa-miR-144-5p	BMP2
hsa-miR-205-5p	BMP3
hsa-miR-144-5p	BMP3
hsa-miR-210-5p	BPIFB1
hsa-miR-205-5p	BTG2
hsa-miR-210-5p	BTG2
hsa-miR-205-5p	C2CD2
hsa-miR-144-5p	C2CD2
hsa-miR-205-5p	C3
hsa-miR-210-5p	C3
hsa-miR-144-5p	C3
hsa-miR-205-5p	C6
hsa-miR-205-5p	C7
hsa-miR-210-5p	C7
hsa-miR-144-5p	C7
hsa-miR-205-5p	CAMK2D
hsa-miR-144-5p	CAMK2D
hsa-miR-210-5p	CASS4
hsa-miR-210-5p	CCDC69
hsa-miR-205-5p	CCR7
hsa-miR-210-5p	CCR7
hsa-miR-144-5p	CCR7
hsa-miR-210-5p	CD14
hsa-miR-144-5p	CD93
hsa-miR-205-5p	CD93
hsa-miR-210-5p	CD93
hsa-miR-205-5p	CDH1
hsa-miR-205-5p	CERCAM
hsa-miR-210-5p	CFI
hsa-miR-210-5p	CLDN3
hsa-miR-205-5p	CNNM1
hsa-miR-205-5p	COLQ
hsa-miR-205-5p	CROT
hsa-miR-205-5p	CRYZ
hsa-miR-144-5p	CRYZ
hsa-miR-205-5p	CTAG2
hsa-miR-205-5p	CYB5A
hsa-miR-205-5p	CYP11A1
hsa-miR-205-5p	DHCR7
hsa-miR-210-5p	DHCR7
hsa-miR-205-5p	DHRS9
hsa-miR-205-5p	DLG5
hsa-miR-10a-5p	DLG5
hsa-miR-205-5p	DUOX2
hsa-miR-205-5p	EGR2
hsa-miR-210-5p	EGR2
hsa-miR-205-5p	EHF
hsa-miR-144-5p	EHF
hsa-miR-210-5p	EHF
hsa-miR-210-5p	EMID1
hsa-miR-205-5p	EPS8

hsa-miR-210-5p	EPS8
hsa-miR-205-5p	FABP3
hsa-miR-205-5p	FADS2
hsa-miR-210-5p	FADS2
hsa-miR-205-5p	FAM102B
hsa-miR-210-5p	FAM102B
hsa-miR-205-5p	FAM118A
hsa-miR-144-5p	FAM118A
hsa-miR-210-5p	FAM118A
hsa-miR-205-5p	FASN
hsa-miR-210-5p	FASN
hsa-miR-205-5p	FBXO32
hsa-miR-144-5p	FBXO32
hsa-miR-210-5p	FBXO32
hsa-miR-205-5p	FCGR3B
hsa-miR-210-5p	FCGR3B
hsa-miR-205-5p	FGD4
hsa-miR-210-5p	FGD4
hsa-miR-144-5p	FGD4
hsa-miR-144-5p	FGF11
hsa-miR-205-5p	FGF11
hsa-miR-210-5p	FGF11
hsa-miR-205-5p	FSTL3
hsa-miR-205-5p	FXYD6
hsa-miR-144-5p	FXYD6
hsa-miR-210-5p	FXYD6
hsa-miR-205-5p	FZD5
hsa-miR-210-5p	FZD5
hsa-miR-210-5p	GALNT1
hsa-miR-205-5p	GALNT1
hsa-miR-10a-5p	GALNT1
hsa-miR-144-5p	GALNT1
hsa-miR-205-5p	GBP5
hsa-miR-205-5p	GNPDA1
hsa-miR-144-5p	GPC4
hsa-miR-205-5p	GPC4
hsa-miR-210-5p	GPC4
hsa-miR-205-5p	GPX3
hsa-miR-210-5p	GPX3
hsa-miR-205-5p	GRIK1
hsa-miR-210-5p	GRIK1
hsa-miR-205-5p	HBEGF
hsa-miR-144-5p	HBEGF
hsa-miR-205-5p	HLA-DQB1
hsa-miR-205-5p	HMGCR
hsa-miR-144-5p	HMGCR
hsa-miR-210-5p	HMGCR
hsa-miR-205-5p	HOOK3
hsa-miR-144-5p	HOOK3
hsa-miR-210-5p	HOOK3
hsa-miR-205-5p	HPS5
hsa-miR-144-5p	HPS5
hsa-miR-205-5p	HPSE
hsa-miR-210-5p	HTRA3
hsa-miR-205-5p	IDE
hsa-miR-205-5p	IDH1
hsa-miR-205-5p	IFIT2
hsa-miR-205-5p	IFITM10

hsa-miR-144-5p	IFITM10
hsa-miR-210-5p	IFITM10
hsa-miR-144-5p	IL10
hsa-miR-205-5p	IL6R
hsa-miR-210-5p	IL6R
hsa-miR-205-5p	INSR
hsa-miR-144-5p	INSR
hsa-miR-205-5p	ITGA9
hsa-miR-210-5p	ITGA9
hsa-miR-144-5p	ITGA9
hsa-miR-144-5p	ITPR1
hsa-miR-210-5p	ITPR1
hsa-miR-205-5p	KCNK3
hsa-miR-210-5p	KCNK3
hsa-miR-205-5p	KCNT2
hsa-miR-144-5p	KCNT2
hsa-miR-210-5p	LDLR
hsa-miR-205-5p	LDLR
hsa-miR-205-5p	LEF1
hsa-miR-210-5p	LEF1
hsa-miR-205-5p	LEFTY2
hsa-miR-205-5p	LGALS12
hsa-miR-210-5p	LIMCH1
hsa-miR-205-5p	LIMCH1
hsa-miR-205-5p	LPAR3
hsa-miR-210-5p	LPAR3
hsa-miR-205-5p	LPIN1
hsa-miR-210-5p	LPIN1
hsa-miR-205-5p	LRAT
hsa-miR-210-5p	LRAT
hsa-miR-210-5p	LRP5
hsa-miR-205-5p	LRRC8C
hsa-miR-144-5p	LRRC8C
hsa-miR-205-5p	LSP1
hsa-miR-205-5p	LSS
hsa-miR-210-5p	LSS
hsa-miR-205-5p	LYZ
hsa-miR-205-5p	MAML2
hsa-miR-210-5p	MAML2
hsa-miR-205-5p	MAP1LC3A
hsa-miR-205-5p	MAP3K5
hsa-miR-205-5p	MAP3K8
hsa-miR-210-5p	MAP3K8
hsa-miR-144-5p	MBNL1
hsa-miR-210-5p	MBNL1
hsa-miR-210-5p	MCM7
hsa-miR-205-5p	MEDAG
hsa-miR-210-5p	MEDAG
hsa-miR-205-5p	MERTK
hsa-miR-205-5p	MGAT5
hsa-miR-210-5p	MGAT5
hsa-miR-205-5p	MMP9
hsa-miR-205-5p	MRO
hsa-miR-210-5p	MRO
hsa-miR-144-5p	MRPS22
hsa-miR-205-5p	MTMR2
hsa-miR-144-5p	MTMR2
hsa-miR-210-5p	MTMR2

hsa-miR-205-5p	MVD
hsa-miR-205-5p	MYO10
hsa-miR-205-5p	MYO5B
hsa-miR-144-5p	MYO5B
hsa-miR-210-5p	MYO5B
hsa-miR-144-5p	NCF1
hsa-miR-205-5p	NCOA4
hsa-miR-144-5p	NCOA4
hsa-miR-205-5p	NDRG2
hsa-miR-144-5p	NDRG2
hsa-miR-210-5p	NDRG2
hsa-miR-144-5p	NELL2
hsa-miR-205-5p	NKAIN1
hsa-miR-210-5p	NKAIN1
hsa-miR-210-5p	NLRP12
hsa-miR-210-5p	NPDC1
hsa-miR-210-5p	NPNT
hsa-miR-205-5p	NPNT
hsa-miR-205-5p	NPY2R
hsa-miR-205-5p	NQO1
hsa-miR-144-5p	NTRK2
hsa-miR-210-5p	NTRK2
hsa-miR-205-5p	OSBPL10
hsa-miR-210-5p	OSBPL10
hsa-miR-205-5p	OSBPL6
hsa-miR-144-5p	OSBPL6
hsa-miR-210-5p	OSBPL6
hsa-miR-205-5p	OSM
hsa-miR-205-5p	OTOF
hsa-miR-210-5p	OTOF
hsa-miR-205-5p	P4HB
hsa-miR-205-5p	PAPSS2
hsa-miR-205-5p	PARD3B
hsa-miR-210-5p	PARD3B
hsa-miR-205-5p	PCSK9
hsa-miR-144-5p	PCSK9
hsa-miR-210-5p	PCSK9
hsa-miR-205-5p	PCYT2
hsa-miR-210-5p	PCYT2
hsa-miR-205-5p	PDK3
hsa-miR-144-5p	PDK3
hsa-miR-210-5p	PDK3
hsa-miR-205-5p	PDZK1IP1
hsa-miR-205-5p	PFKFB4
hsa-miR-210-5p	PFKFB4
hsa-miR-210-5p	PHACTR4
hsa-miR-210-5p	PHKA2
hsa-miR-205-5p	PIGR
hsa-miR-210-5p	PIM1
hsa-miR-205-5p	PINX1
hsa-miR-210-5p	PINX1
hsa-miR-205-5p	PLAT
hsa-miR-205-5p	PLP1
hsa-miR-210-5p	PLP1
hsa-miR-205-5p	PMAIP1
hsa-miR-205-5p	PMEPA1
hsa-miR-144-5p	PMEPA1
hsa-miR-210-5p	PMEPA1

hsa-miR-210-5p	PNCK
hsa-miR-205-5p	PNPLA3
hsa-miR-210-5p	PNPLA3
hsa-miR-144-5p	POLR1B
hsa-miR-205-5p	PPP1R12B
hsa-miR-144-5p	PPP1R12B
hsa-miR-210-5p	PPP1R12B
hsa-miR-205-5p	PRDX3
hsa-miR-205-5p	PRKCZ
hsa-miR-205-5p	PRLR
hsa-miR-210-5p	PRLR
hsa-miR-144-5p	PRLR
hsa-miR-205-5p	PRND
hsa-miR-210-5p	PRND
hsa-miR-205-5p	PRUNE2
hsa-miR-144-5p	PRUNE2
hsa-miR-205-5p	PTPN13
hsa-miR-144-5p	QPRT
hsa-miR-205-5p	QPRT
hsa-miR-144-5p	RALGAPA2
hsa-miR-205-5p	REPS2
hsa-miR-210-5p	REPS2
hsa-miR-144-5p	REPS2
hsa-miR-205-5p	RGS12
hsa-miR-210-5p	S100A8
hsa-miR-210-5p	SCARB1
hsa-miR-205-5p	SCD
hsa-miR-210-5p	SCD
hsa-miR-205-5p	SCN3B
hsa-miR-144-5p	SCN3B
hsa-miR-210-5p	SCN3B
hsa-miR-205-5p	SEC14L2
hsa-miR-210-5p	SEC14L2
hsa-miR-210-5p	SERINC5
hsa-miR-205-5p	SERINC5
hsa-miR-144-5p	SERINC5
hsa-miR-205-5p	SERPINA1
hsa-miR-144-5p	SERPINA1
hsa-miR-144-5p	SERPINA5
hsa-miR-205-5p	SERPINA5
hsa-miR-205-5p	SERPINB2
hsa-miR-210-5p	SH2D3C
hsa-miR-210-5p	SIL1
hsa-miR-210-5p	SLC2A6
hsa-miR-205-5p	SLC2A6
hsa-miR-205-5p	SLC40A1
hsa-miR-210-5p	SLC6A8
hsa-miR-210-5p	SLC7A4
hsa-miR-205-5p	SLC9A7
hsa-miR-10a-5p	SLC9A7
hsa-miR-144-5p	SLC9A7
hsa-miR-210-5p	SLC9A7
hsa-miR-144-5p	SLPI
hsa-miR-144-5p	SOBP
hsa-miR-10a-5p	SOBP
hsa-miR-210-5p	SOBP
hsa-miR-205-5p	SOCS3
hsa-miR-210-5p	SOCS3

hsa-miR-144-5p	SOD2
hsa-miR-205-5p	SOD2
hsa-miR-210-5p	SOD2
hsa-miR-205-5p	SPOCK3
hsa-miR-210-5p	SPOCK3
hsa-miR-144-5p	SPOCK3
hsa-miR-144-5p	ST6GAL2
hsa-miR-205-5p	ST6GAL2
hsa-miR-210-5p	ST6GAL2
hsa-miR-205-5p	STC1
hsa-miR-144-5p	STC1
hsa-miR-210-5p	STC1
hsa-miR-205-5p	STON1
hsa-miR-144-5p	STON1
hsa-miR-210-5p	STON1
hsa-miR-210-5p	STRADB
hsa-miR-205-5p	SV2C
hsa-miR-144-5p	SV2C
hsa-miR-210-5p	SV2C
hsa-miR-210-5p	SYNE2
hsa-miR-205-5p	TACSTD2
hsa-miR-144-5p	TAP2
hsa-miR-210-5p	TAP2
hsa-miR-205-5p	TBC1D10C
hsa-miR-210-5p	TBC1D10C
hsa-miR-210-5p	TBC1D22A
hsa-miR-205-5p	TBC1D9B
hsa-miR-205-5p	TFPI2
hsa-miR-210-5p	TFPI2
hsa-miR-205-5p	THSD7A
hsa-miR-144-5p	THSD7A
hsa-miR-205-5p	TIMMDC1
hsa-miR-210-5p	TIMMDC1
hsa-miR-205-5p	TNFRSF25
hsa-miR-210-5p	TNFRSF25
hsa-miR-205-5p	TP53INP2
hsa-miR-210-5p	TP53INP2
hsa-miR-205-5p	TREM1
hsa-miR-144-5p	TREM1
hsa-miR-210-5p	TREM1
hsa-miR-205-5p	TSHZ2
hsa-miR-144-5p	TSHZ2
hsa-miR-210-5p	TSHZ2
hsa-miR-205-5p	UBE2QL1
hsa-miR-210-5p	UBE2QL1
hsa-miR-144-5p	VCAN
hsa-miR-205-5p	VCAN
hsa-miR-210-5p	VCAN
hsa-miR-210-5p	ZDBF2
hsa-miR-205-5p	ZNF395
hsa-miR-210-5p	ZNF395
hsa-miR-205-5p	ZSCAN1
hsa-miR-210-5p	ZSCAN1
