# Supplementary material for: Integrated bioinformatics analysis elucidates granulosa cell whole-transcriptome landscape of PCOS in China
Source: J Ovarian Res. 2023 Aug 3;16:154. doi: 10.1186/s13048-023-01223-0 (PMC10398987; doi:10.1186/s13048-023-01223-0)
Supplement: Supplementary file 2 — Additional file 2: Supplemental Table 2. The circRNA-miRNA-mRNA Network. [file 13048_2023_1223_MOESM2_ESM.pdf]

The circRNA-miRNA-mRNA Network

miRNA	name
hsa-miR-877-5p	hsa_circ_0038634
hsa-miR-205-5p	hsa_circ_0064210
hsa-miR-877-5p	hsa_circ_0079022
hsa-miR-205-5p	hsa_circ_0107605
hsa-miR-205-5p	hsa_circ_0029118
hsa-miR-205-5p	hsa_circ_0045707
hsa-miR-205-5p	hsa_circ_0063556
hsa-miR-205-5p	hsa_circ_0016457
hsa-miR-205-5p	hsa_circ_0027652
hsa-miR-205-5p	hsa_circ_0027645
hsa-miR-205-5p	hsa_circ_0087188
hsa-miR-205-5p	hsa_circ_0028682
hsa-miR-877-5p	hsa_circ_0101220
hsa-miR-877-5p	hsa_circ_0038633
hsa-miR-205-5p	hsa_circ_0077696
hsa-miR-877-5p	hsa_circ_0049720
hsa-miR-205-5p	hsa_circ_0139585
hsa-miR-205-5p	hsa_circ_0019227
hsa-miR-205-5p	hsa_circ_0101210
hsa-miR-205-5p	hsa_circ_0038387
hsa-miR-205-5p	hsa_circ_0102806
hsa-miR-205-5p	hsa_circ_0101220
hsa-miR-877-5p	hsa_circ_0124441
hsa-miR-877-5p	hsa_circ_0065460
hsa-miR-205-5p	hsa_circ_0118448
hsa-miR-205-5p	hsa_circ_0139587
hsa-miR-205-5p	hsa_circ_0080631
hsa-miR-205-5p	hsa_circ_0111332
hsa-miR-877-5p	hsa_circ_0083383
hsa-miR-205-5p	hsa_circ_0020555
hsa-miR-205-5p	hsa_circ_0102812
hsa-miR-205-5p	hsa_circ_0032856
hsa-miR-205-5p	hsa_circ_0073639
hsa-miR-205-5p	hsa_circ_0086809
hsa-miR-205-5p	hsa_circ_0125022
hsa-miR-205-5p	hsa_circ_0069663
hsa-miR-205-5p	hsa_circ_0100972
hsa-miR-205-5p	hsa_circ_0015772
hsa-miR-877-5p	hsa_circ_0078783
hsa-miR-877-5p	hsa_circ_0009945
hsa-miR-205-5p	hsa_circ_0114633
hsa-miR-205-5p	hsa_circ_0131048
hsa-miR-205-5p	hsa_circ_0027651
hsa-miR-877-5p	hsa_circ_0115243
hsa-miR-877-5p	hsa_circ_0101210
hsa-miR-205-5p	hsa_circ_0136942
hsa-miR-877-5p	hsa_circ_0020555
hsa-miR-877-5p	hsa_circ_0016068
hsa-miR-877-5p	hsa_circ_0016457
hsa-miR-100-3p	hsa_circ_0077696
hsa-miR-144-5p	hsa_circ_0122399
hsa-miR-100-3p	hsa_circ_0132400
hsa-miR-100-3p	hsa_circ_0027652
hsa-miR-100-3p	hsa_circ_0107925
hsa-miR-100-3p	hsa_circ_0027651
hsa-miR-100-3p	hsa_circ_0066945

hsa-miR-100-3p	hsa_circ_0027645
hsa-miR-100-3p	hsa_circ_0059550
hsa-miR-100-3p	hsa_circ_0024058
hsa-miR-144-5p	hsa_circ_0015411
hsa-miR-100-3p	hsa_circ_0019227
hsa-miR-100-3p	hsa_circ_0126755
hsa-miR-100-3p	hsa_circ_0073349
hsa-miR-210-5p	hsa_circ_0066945
hsa-miR-144-5p	hsa_circ_0079877
hsa-miR-210-5p	hsa_circ_0045707
hsa-miR-100-3p	hsa_circ_0107923
hsa-miR-144-5p	hsa_circ_0019164
hsa-miR-100-3p	hsa_circ_0097067
hsa-miR-144-5p	hsa_circ_0107923
hsa-miR-144-5p	hsa_circ_0027651
hsa-miR-210-5p	hsa_circ_0025425
hsa-miR-100-3p	hsa_circ_0038387
hsa-miR-144-5p	hsa_circ_0073385
hsa-miR-210-5p	hsa_circ_0027788
hsa-miR-100-3p	hsa_circ_0114633
hsa-miR-210-5p	hsa_circ_0015866
hsa-miR-210-5p	hsa_circ_0080653
hsa-miR-100-3p	hsa_circ_0107605
hsa-miR-100-3p	hsa_circ_0015772
hsa-miR-144-5p	hsa_circ_0027652
hsa-miR-144-5p	hsa_circ_0107925
hsa-miR-144-5p	hsa_circ_0026778
hsa-miR-144-5p	hsa_circ_0077696
hsa-miR-144-5p	hsa_circ_0020060
hsa-miR-210-5p	hsa_circ_0103220
hsa-miR-210-5p	hsa_circ_0037694
hsa-miR-100-3p	hsa_circ_0034695
hsa-miR-100-3p	hsa_circ_0086581
hsa-miR-144-5p	hsa_circ_0125357
hsa-miR-144-5p	hsa_circ_0032856
hsa-miR-144-5p	hsa_circ_0086535
hsa-miR-144-5p	hsa_circ_0027645
hsa-miR-144-5p	hsa_circ_0114633
hsa-miR-144-5p	hsa_circ_0100972
hsa-miR-210-5p	hsa_circ_0009945
hsa-miR-210-5p	hsa_circ_0020792
hsa-miR-210-5p	hsa_circ_0024421
hsa-miR-210-5p	hsa_circ_0023399
hsa-miR-210-5p	hsa_circ_0105454
hsa-miR-144-5p	hsa_circ_0067739
hsa-miR-144-5p	hsa_circ_0080444
hsa-miR-144-5p	hsa_circ_0050847
hsa-miR-144-5p	hsa_circ_0028018
hsa-miR-100-3p	hsa_circ_0126754
hsa-miR-144-5p	hsa_circ_0047019
hsa-miR-144-5p	hsa_circ_0047008
hsa-miR-100-3p	hsa_circ_0137422
hsa-miR-144-5p	hsa_circ_0070981
hsa-miR-100-3p	hsa_circ_0013210
hsa-miR-144-5p	hsa_circ_0073349
hsa-miR-144-5p	hsa_circ_0020058
hsa-miR-10a-5p	hsa_circ_0020060
hsa-miR-10a-5p	hsa_circ_0020058

hsa-miR-10a-5p	hsa_circ_0087188
hsa-miR-10a-5p	hsa_circ_0013210
hsa-miR-10a-5p	hsa_circ_0074193
hsa-miR-10a-5p	hsa_circ_0050847
hsa-miR-10a-5p	hsa_circ_0112444
hsa-miR-10a-5p	hsa_circ_0073385
hsa-miR-10a-5p	hsa_circ_0086535
hsa-miR-10a-5p	hsa_circ_0064210
hsa-miR-10a-5p	hsa_circ_0086809
hsa-miR-877-5p	AACS
hsa-miR-205-5p	AACS
hsa-miR-205-5p	ACO1
hsa-miR-100-3p	ACO1
hsa-miR-144-5p	ACO1
hsa-miR-877-5p	ACO1
hsa-miR-210-5p	ACO1
hsa-miR-144-5p	ACSM1
hsa-miR-205-5p	ACSS2
hsa-miR-210-5p	ACSS2
hsa-miR-877-5p	ACSS2
hsa-miR-205-5p	ACSS3
hsa-miR-100-3p	ACSS3
hsa-miR-205-5p	ADAMTS4
hsa-miR-100-3p	ADAMTS4
hsa-miR-877-5p	ADAMTS4
hsa-miR-210-5p	ADAMTS4
hsa-miR-877-5p	AFF3
hsa-miR-205-5p	AFF3
hsa-miR-210-5p	AFF3
hsa-miR-100-3p	AFF3
hsa-miR-205-5p	AGFG2
hsa-miR-210-5p	AGFG2
hsa-miR-100-3p	AGFG2
hsa-miR-877-5p	AGFG2
hsa-miR-210-5p	AK7
hsa-miR-205-5p	AKAP5
hsa-miR-100-3p	AKAP5
hsa-miR-877-5p	AKAP5
hsa-miR-144-5p	AKAP5
hsa-miR-210-5p	AKAP5
hsa-miR-877-5p	APOE
hsa-miR-877-5p	ARHGAP9
hsa-miR-877-5p	ARID5A
hsa-miR-205-5p	ARID5A
hsa-miR-210-5p	ARID5A
hsa-miR-205-5p	ATF3
hsa-miR-144-5p	ATF3
hsa-miR-877-5p	ATOH8
hsa-miR-100-3p	ATOH8
hsa-miR-205-5p	ATOH8
hsa-miR-210-5p	ATOH8
hsa-miR-877-5p	ATP10D
hsa-miR-877-5p	BMP2
hsa-miR-205-5p	BMP2
hsa-miR-144-5p	BMP2
hsa-miR-210-5p	BMP2
hsa-miR-100-3p	BMP2
hsa-miR-100-3p	BMP3

hsa-miR-205-5p	BMP3
hsa-miR-144-5p	BMP3
hsa-miR-877-5p	BMP3
hsa-miR-210-5p	BPIFB1
hsa-miR-205-5p	BTG2
hsa-miR-210-5p	BTG2
hsa-miR-877-5p	BTG2
hsa-miR-877-5p	C1orf141
hsa-miR-877-5p	C2CD2
hsa-miR-144-5p	C2CD2
hsa-miR-100-3p	C2CD2
hsa-miR-205-5p	C2CD2
hsa-miR-210-5p	C3
hsa-miR-877-5p	C3
hsa-miR-205-5p	C3
hsa-miR-100-3p	C3
hsa-miR-144-5p	C3
hsa-miR-205-5p	C6
hsa-miR-877-5p	C6
hsa-miR-100-3p	C6
hsa-miR-205-5p	C7
hsa-miR-144-5p	C7
hsa-miR-210-5p	C7
hsa-miR-877-5p	C7
hsa-miR-205-5p	CAMK2D
hsa-miR-144-5p	CAMK2D
hsa-miR-100-3p	CAMK2D
hsa-miR-877-5p	CAMK2D
hsa-miR-210-5p	CASS4
hsa-miR-100-3p	CCDC69
hsa-miR-877-5p	CCDC69
hsa-miR-210-5p	CCDC69
hsa-miR-144-5p	CCR7
hsa-miR-210-5p	CCR7
hsa-miR-205-5p	CCR7
hsa-miR-877-5p	CCR7
hsa-miR-100-3p	CCR7
hsa-miR-210-5p	CD14
hsa-miR-205-5p	CD93
hsa-miR-877-5p	CD93
hsa-miR-144-5p	CD93
hsa-miR-100-3p	CD93
hsa-miR-210-5p	CD93
hsa-miR-100-3p	CDH1
hsa-miR-205-5p	CDH1
hsa-miR-205-5p	CERCAM
hsa-miR-210-5p	CFI
hsa-miR-210-5p	CLDN3
hsa-miR-877-5p	CNNM1
hsa-miR-100-3p	CNNM1
hsa-miR-205-5p	CNNM1
hsa-miR-205-5p	COLQ
hsa-miR-100-3p	COLQ
hsa-miR-205-5p	CROT
hsa-miR-144-5p	CRYZ
hsa-miR-205-5p	CRYZ
hsa-miR-100-3p	CRYZ
hsa-miR-877-5p	CRYZ

hsa-miR-205-5p	CTAG2
hsa-miR-100-3p	CYB5A
hsa-miR-205-5p	CYB5A
hsa-miR-205-5p	CYP11A1
hsa-miR-100-3p	CYP11A1
hsa-miR-205-5p	DHCR7
hsa-miR-210-5p	DHCR7
hsa-miR-205-5p	DHRS9
hsa-miR-100-3p	DLG5
hsa-miR-205-5p	DLG5
hsa-miR-10a-5p	DLG5
hsa-miR-877-5p	DNER
hsa-miR-100-3p	DNER
hsa-miR-205-5p	DUOX2
hsa-miR-205-5p	EGR2
hsa-miR-210-5p	EGR2
hsa-miR-144-5p	EHF
hsa-miR-205-5p	EHF
hsa-miR-877-5p	EHF
hsa-miR-210-5p	EHF
hsa-miR-210-5p	EMID1
hsa-miR-877-5p	ENO2
hsa-miR-205-5p	EPS8
hsa-miR-100-3p	EPS8
hsa-miR-210-5p	EPS8
hsa-miR-205-5p	FABP3
hsa-miR-205-5p	FADS2
hsa-miR-210-5p	FADS2
hsa-miR-877-5p	FAM102B
hsa-miR-205-5p	FAM102B
hsa-miR-210-5p	FAM102B
hsa-miR-210-5p	FAM118A
hsa-miR-205-5p	FAM118A
hsa-miR-100-3p	FAM118A
hsa-miR-144-5p	FAM118A
hsa-miR-877-5p	FAM118A
hsa-miR-210-5p	FASN
hsa-miR-205-5p	FASN
hsa-miR-205-5p	FBXO32
hsa-miR-100-3p	FBXO32
hsa-miR-877-5p	FBXO32
hsa-miR-144-5p	FBXO32
hsa-miR-210-5p	FBXO32
hsa-miR-100-3p	FCGR3B
hsa-miR-210-5p	FCGR3B
hsa-miR-205-5p	FCGR3B
hsa-miR-877-5p	FCGR3B
hsa-miR-205-5p	FGD4
hsa-miR-877-5p	FGD4
hsa-miR-100-3p	FGD4
hsa-miR-144-5p	FGD4
hsa-miR-210-5p	FGD4
hsa-miR-144-5p	FGF11
hsa-miR-877-5p	FGF11
hsa-miR-100-3p	FGF11
hsa-miR-205-5p	FGF11
hsa-miR-210-5p	FGF11
hsa-miR-205-5p	FSTL3

hsa-miR-100-3p	FSTL3
hsa-miR-205-5p	FXYD6
hsa-miR-877-5p	FXYD6
hsa-miR-210-5p	FXYD6
hsa-miR-144-5p	FXYD6
hsa-miR-210-5p	FZD5
hsa-miR-205-5p	FZD5
hsa-miR-877-5p	FZD5
hsa-miR-100-3p	FZD5
hsa-miR-144-5p	GALNT1
hsa-miR-210-5p	GALNT1
hsa-miR-205-5p	GALNT1
hsa-miR-10a-5p	GALNT1
hsa-miR-100-3p	GALNT1
hsa-miR-877-5p	GALNT1
hsa-miR-205-5p	GBP5
hsa-miR-877-5p	GBP5
hsa-miR-205-5p	GNPDA1
hsa-miR-205-5p	GPC4
hsa-miR-877-5p	GPC4
hsa-miR-144-5p	GPC4
hsa-miR-100-3p	GPC4
hsa-miR-210-5p	GPC4
hsa-miR-210-5p	GPX3
hsa-miR-205-5p	GPX3
hsa-miR-100-3p	GPX3
hsa-miR-210-5p	GRIK1
hsa-miR-205-5p	GRIK1
hsa-miR-100-3p	GRIK1
hsa-miR-144-5p	HBEGF
hsa-miR-205-5p	HBEGF
hsa-miR-877-5p	HBEGF
hsa-miR-205-5p	HLA-DQB1
hsa-miR-144-5p	HMGCR
hsa-miR-210-5p	HMGCR
hsa-miR-205-5p	HMGCR
hsa-miR-100-3p	HMGCR
hsa-miR-877-5p	HMGCR
hsa-miR-877-5p	HOOK3
hsa-miR-100-3p	HOOK3
hsa-miR-205-5p	HOOK3
hsa-miR-144-5p	HOOK3
hsa-miR-210-5p	HOOK3
hsa-miR-205-5p	HPS5
hsa-miR-144-5p	HPS5
hsa-miR-100-3p	HPSE
hsa-miR-205-5p	HPSE
hsa-miR-877-5p	HPSE
hsa-miR-100-3p	HTRA3
hsa-miR-210-5p	HTRA3
hsa-miR-205-5p	IDE
hsa-miR-100-3p	IDE
hsa-miR-877-5p	IDE
hsa-miR-100-3p	IDH1
hsa-miR-205-5p	IDH1
hsa-miR-205-5p	IFIT2
hsa-miR-100-3p	IFIT2
hsa-miR-205-5p	IFITM10

hsa-miR-144-5p	IFITM10
hsa-miR-210-5p	IFITM10
hsa-miR-877-5p	IL10
hsa-miR-144-5p	IL10
hsa-miR-205-5p	IL6R
hsa-miR-100-3p	IL6R
hsa-miR-210-5p	IL6R
hsa-miR-100-3p	INSR
hsa-miR-205-5p	INSR
hsa-miR-144-5p	INSR
hsa-miR-210-5p	ITGA9
hsa-miR-205-5p	ITGA9
hsa-miR-144-5p	ITGA9
hsa-miR-100-3p	ITGA9
hsa-miR-877-5p	ITGA9
hsa-miR-100-3p	ITPR1
hsa-miR-144-5p	ITPR1
hsa-miR-210-5p	ITPR1
hsa-miR-877-5p	ITPR1
hsa-miR-205-5p	KCNK3
hsa-miR-877-5p	KCNK3
hsa-miR-210-5p	KCNK3
hsa-miR-205-5p	KCNT2
hsa-miR-144-5p	KCNT2
hsa-miR-205-5p	LDLR
hsa-miR-210-5p	LDLR
hsa-miR-100-3p	LDLR
hsa-miR-100-3p	LEF1
hsa-miR-877-5p	LEF1
hsa-miR-205-5p	LEF1
hsa-miR-210-5p	LEF1
hsa-miR-877-5p	LEFTY2
hsa-miR-205-5p	LEFTY2
hsa-miR-100-3p	LEFTY2
hsa-miR-205-5p	LGALS12
hsa-miR-877-5p	LGALS12
hsa-miR-205-5p	LIMCH1
hsa-miR-210-5p	LIMCH1
hsa-miR-205-5p	LPAR3
hsa-miR-210-5p	LPAR3
hsa-miR-100-3p	LPAR3
hsa-miR-877-5p	LPIN1
hsa-miR-205-5p	LPIN1
hsa-miR-100-3p	LPIN1
hsa-miR-210-5p	LPIN1
hsa-miR-205-5p	LRAT
hsa-miR-210-5p	LRAT
hsa-miR-100-3p	LRAT
hsa-miR-210-5p	LRP5
hsa-miR-144-5p	LRRC8C
hsa-miR-877-5p	LRRC8C
hsa-miR-205-5p	LRRC8C
hsa-miR-100-3p	LRRC8C
hsa-miR-205-5p	LSP1
hsa-miR-205-5p	LSS
hsa-miR-210-5p	LSS
hsa-miR-205-5p	LYZ
hsa-miR-205-5p	MAML2

hsa-miR-100-3p	MAML2
hsa-miR-877-5p	MAML2
hsa-miR-210-5p	MAML2
hsa-miR-205-5p	MAP1LC3A
hsa-miR-205-5p	MAP3K5
hsa-miR-100-3p	MAP3K8
hsa-miR-205-5p	MAP3K8
hsa-miR-210-5p	MAP3K8
hsa-miR-144-5p	MBNL1
hsa-miR-210-5p	MBNL1
hsa-miR-210-5p	MCM7
hsa-miR-210-5p	MEDAG
hsa-miR-205-5p	MEDAG
hsa-miR-205-5p	MERTK
hsa-miR-205-5p	MGAT5
hsa-miR-210-5p	MGAT5
hsa-miR-205-5p	MMP9
hsa-miR-100-3p	MRO
hsa-miR-205-5p	MRO
hsa-miR-877-5p	MRO
hsa-miR-210-5p	MRO
hsa-miR-144-5p	MRPS22
hsa-miR-144-5p	MTMR2
hsa-miR-205-5p	MTMR2
hsa-miR-877-5p	MTMR2
hsa-miR-100-3p	MTMR2
hsa-miR-210-5p	MTMR2
hsa-miR-100-3p	MUC5B
hsa-miR-205-5p	MVD
hsa-miR-877-5p	MVD
hsa-miR-205-5p	MYO10
hsa-miR-877-5p	MYO10
hsa-miR-100-3p	MYO10
hsa-miR-210-5p	MYO5B
hsa-miR-144-5p	MYO5B
hsa-miR-205-5p	MYO5B
hsa-miR-100-3p	MYO5B
hsa-miR-144-5p	NCF1
hsa-miR-877-5p	NCF1
hsa-miR-205-5p	NCOA4
hsa-miR-144-5p	NCOA4
hsa-miR-877-5p	NCOA4
hsa-miR-100-3p	NCOA4
hsa-miR-144-5p	NDRG2
hsa-miR-205-5p	NDRG2
hsa-miR-877-5p	NDRG2
hsa-miR-210-5p	NDRG2
hsa-miR-144-5p	NELL2
hsa-miR-877-5p	NELL2
hsa-miR-100-3p	NELL2
hsa-miR-205-5p	NKAIN1
hsa-miR-877-5p	NKAIN1
hsa-miR-210-5p	NKAIN1
hsa-miR-100-3p	NLRP12
hsa-miR-210-5p	NLRP12
hsa-miR-100-3p	NPAS2
hsa-miR-210-5p	NPDC1
hsa-miR-205-5p	NPNT

hsa-miR-210-5p	NPNT
hsa-miR-205-5p	NPY2R
hsa-miR-205-5p	NQO1
hsa-miR-877-5p	NR1H4
hsa-miR-144-5p	NTRK2
hsa-miR-210-5p	NTRK2
hsa-miR-100-3p	NTRK2
hsa-miR-205-5p	OSBPL10
hsa-miR-210-5p	OSBPL10
hsa-miR-100-3p	OSBPL10
hsa-miR-877-5p	OSBPL6
hsa-miR-205-5p	OSBPL6
hsa-miR-100-3p	OSBPL6
hsa-miR-144-5p	OSBPL6
hsa-miR-210-5p	OSBPL6
hsa-miR-205-5p	OSM
hsa-miR-877-5p	OSM
hsa-miR-205-5p	OTOF
hsa-miR-210-5p	OTOF
hsa-miR-877-5p	OTOF
hsa-miR-205-5p	P4HB
hsa-miR-205-5p	PAPSS2
hsa-miR-100-3p	PARD3B
hsa-miR-877-5p	PARD3B
hsa-miR-205-5p	PARD3B
hsa-miR-210-5p	PARD3B
hsa-miR-205-5p	PCSK9
hsa-miR-144-5p	PCSK9
hsa-miR-210-5p	PCSK9
hsa-miR-877-5p	PCSK9
hsa-miR-100-3p	PCYT2
hsa-miR-210-5p	PCYT2
hsa-miR-205-5p	PCYT2
hsa-miR-877-5p	PDK3
hsa-miR-100-3p	PDK3
hsa-miR-205-5p	PDK3
hsa-miR-210-5p	PDK3
hsa-miR-144-5p	PDK3
hsa-miR-205-5p	PDZK1IP1
hsa-miR-205-5p	PFKFB4
hsa-miR-100-3p	PFKFB4
hsa-miR-877-5p	PFKFB4
hsa-miR-210-5p	PFKFB4
hsa-miR-100-3p	PHACTR4
hsa-miR-210-5p	PHACTR4
hsa-miR-877-5p	PHACTR4
hsa-miR-877-5p	PHF1
hsa-miR-210-5p	PHKA2
hsa-miR-877-5p	PHKA2
hsa-miR-205-5p	PIGR
hsa-miR-877-5p	PIGR
hsa-miR-100-3p	PIGR
hsa-miR-100-3p	PIM1
hsa-miR-210-5p	PIM1
hsa-miR-205-5p	PINX1
hsa-miR-210-5p	PINX1
hsa-miR-205-5p	PLAT
hsa-miR-877-5p	PLAT

hsa-miR-205-5p	PLP1
hsa-miR-210-5p	PLP1
hsa-miR-205-5p	PMAIP1
hsa-miR-205-5p	PMEPA1
hsa-miR-100-3p	PMEPA1
hsa-miR-877-5p	PMEPA1
hsa-miR-210-5p	PMEPA1
hsa-miR-144-5p	PMEPA1
hsa-miR-210-5p	PNCK
hsa-miR-205-5p	PNPLA3
hsa-miR-210-5p	PNPLA3
hsa-miR-877-5p	PNPLA3
hsa-miR-877-5p	POLR1B
hsa-miR-144-5p	POLR1B
hsa-miR-877-5p	PPP1R12B
hsa-miR-205-5p	PPP1R12B
hsa-miR-100-3p	PPP1R12B
hsa-miR-144-5p	PPP1R12B
hsa-miR-210-5p	PPP1R12B
hsa-miR-205-5p	PRDX3
hsa-miR-205-5p	PRKCZ
hsa-miR-100-3p	PRLR
hsa-miR-144-5p	PRLR
hsa-miR-877-5p	PRLR
hsa-miR-205-5p	PRLR
hsa-miR-210-5p	PRLR
hsa-miR-205-5p	PRND
hsa-miR-877-5p	PRND
hsa-miR-210-5p	PRND
hsa-miR-205-5p	PRUNE2
hsa-miR-100-3p	PRUNE2
hsa-miR-144-5p	PRUNE2
hsa-miR-205-5p	PTPN13
hsa-miR-877-5p	PTPN13
hsa-miR-205-5p	QPRT
hsa-miR-144-5p	QPRT
hsa-miR-877-5p	RAC2
hsa-miR-100-3p	RALGAPA2
hsa-miR-877-5p	RALGAPA2
hsa-miR-144-5p	RALGAPA2
hsa-miR-205-5p	REPS2
hsa-miR-144-5p	REPS2
hsa-miR-877-5p	REPS2
hsa-miR-210-5p	REPS2
hsa-miR-100-3p	REPS2
hsa-miR-205-5p	RGS12
hsa-miR-100-3p	RGS12
hsa-miR-210-5p	S100A8
hsa-miR-210-5p	SCARB1
hsa-miR-100-3p	SCARB1
hsa-miR-205-5p	SCD
hsa-miR-210-5p	SCD
hsa-miR-877-5p	SCD
hsa-miR-144-5p	SCN3B
hsa-miR-205-5p	SCN3B
hsa-miR-877-5p	SCN3B
hsa-miR-100-3p	SCN3B
hsa-miR-210-5p	SCN3B

hsa-miR-205-5p	SEC14L2
hsa-miR-877-5p	SEC14L2
hsa-miR-210-5p	SEC14L2
hsa-miR-100-3p	SEMA4A
hsa-miR-205-5p	SERINC5
hsa-miR-877-5p	SERINC5
hsa-miR-100-3p	SERINC5
hsa-miR-144-5p	SERINC5
hsa-miR-210-5p	SERINC5
hsa-miR-144-5p	SERPINA1
hsa-miR-205-5p	SERPINA1
hsa-miR-877-5p	SERPINA1
hsa-miR-100-3p	SERPINA1
hsa-miR-205-5p	SERPINA5
hsa-miR-144-5p	SERPINA5
hsa-miR-205-5p	SERPINB2
hsa-miR-210-5p	SH2D3C
hsa-miR-210-5p	SIL1
hsa-miR-205-5p	SLC2A6
hsa-miR-210-5p	SLC2A6
hsa-miR-205-5p	SLC40A1
hsa-miR-100-3p	SLC40A1
hsa-miR-100-3p	SLC6A8
hsa-miR-210-5p	SLC6A8
hsa-miR-210-5p	SLC7A4
hsa-miR-100-3p	SLC7A4
hsa-miR-144-5p	SLC9A7
hsa-miR-210-5p	SLC9A7
hsa-miR-205-5p	SLC9A7
hsa-miR-10a-5p	SLC9A7
hsa-miR-100-3p	SLC9A7
hsa-miR-144-5p	SLPI
hsa-miR-144-5p	SOBP
hsa-miR-210-5p	SOBP
hsa-miR-10a-5p	SOBP
hsa-miR-100-3p	SOBP
hsa-miR-877-5p	SOBP
hsa-miR-205-5p	SOCS3
hsa-miR-210-5p	SOCS3
hsa-miR-144-5p	SOD2
hsa-miR-205-5p	SOD2
hsa-miR-877-5p	SOD2
hsa-miR-100-3p	SOD2
hsa-miR-210-5p	SOD2
hsa-miR-205-5p	SPOCK3
hsa-miR-100-3p	SPOCK3
hsa-miR-144-5p	SPOCK3
hsa-miR-210-5p	SPOCK3
hsa-miR-205-5p	ST6GAL2
hsa-miR-877-5p	ST6GAL2
hsa-miR-144-5p	ST6GAL2
hsa-miR-100-3p	ST6GAL2
hsa-miR-210-5p	ST6GAL2
hsa-miR-210-5p	STC1
hsa-miR-205-5p	STC1
hsa-miR-100-3p	STC1
hsa-miR-144-5p	STC1
hsa-miR-877-5p	STC1

hsa-miR-205-5p	STON1
hsa-miR-144-5p	STON1
hsa-miR-877-5p	STON1
hsa-miR-100-3p	STON1
hsa-miR-210-5p	STON1
hsa-miR-877-5p	STRADB
hsa-miR-210-5p	STRADB
hsa-miR-205-5p	SV2C
hsa-miR-100-3p	SV2C
hsa-miR-877-5p	SV2C
hsa-miR-144-5p	SV2C
hsa-miR-210-5p	SV2C
hsa-miR-210-5p	SYNE2
hsa-miR-205-5p	TACSTD2
hsa-miR-100-3p	TAP2
hsa-miR-144-5p	TAP2
hsa-miR-877-5p	TAP2
hsa-miR-210-5p	TAP2
hsa-miR-205-5p	TAP2
hsa-miR-205-5p	TBC1D10C
hsa-miR-210-5p	TBC1D10C
hsa-miR-877-5p	TBC1D10C
hsa-miR-210-5p	TBC1D22A
hsa-miR-205-5p	TBC1D9B
hsa-miR-877-5p	TBC1D9B
hsa-miR-877-5p	TFF3
hsa-miR-210-5p	TFPI2
hsa-miR-205-5p	TFPI2
hsa-miR-877-5p	THBD
hsa-miR-144-5p	THSD7A
hsa-miR-205-5p	THSD7A
hsa-miR-877-5p	THSD7A
hsa-miR-100-3p	THSD7A
hsa-miR-877-5p	TIMMDC1
hsa-miR-205-5p	TIMMDC1
hsa-miR-210-5p	TIMMDC1
hsa-miR-100-3p	TM7SF2
hsa-miR-877-5p	TNFRSF25
hsa-miR-205-5p	TNFRSF25
hsa-miR-210-5p	TNFRSF25
hsa-miR-100-3p	TP53INP2
hsa-miR-210-5p	TP53INP2
hsa-miR-877-5p	TP53INP2
hsa-miR-205-5p	TP53INP2
hsa-miR-205-5p	TREM1
hsa-miR-877-5p	TREM1
hsa-miR-100-3p	TREM1
hsa-miR-144-5p	TREM1
hsa-miR-210-5p	TREM1
hsa-miR-205-5p	TSHZ2
hsa-miR-877-5p	TSHZ2
hsa-miR-144-5p	TSHZ2
hsa-miR-100-3p	TSHZ2
hsa-miR-210-5p	TSHZ2
hsa-miR-210-5p	UBE2QL1
hsa-miR-205-5p	UBE2QL1
hsa-miR-877-5p	UBE2QL1
hsa-miR-100-3p	UBE2QL1

hsa-miR-877-5p	UPK1A
hsa-miR-100-3p	VCAM1
hsa-miR-205-5p	VCAN
hsa-miR-144-5p	VCAN
hsa-miR-100-3p	VCAN
hsa-miR-210-5p	VCAN
hsa-miR-210-5p	ZDBF2
hsa-miR-100-3p	ZDBF2
hsa-miR-205-5p	ZNF395
hsa-miR-100-3p	ZNF395
hsa-miR-210-5p	ZNF395
hsa-miR-205-5p	ZSCAN1
hsa-miR-210-5p	ZSCAN1
