# Supplementary material for: Integrated bioinformatics analysis elucidates granulosa cell whole-transcriptome landscape of PCOS in China
Source: J Ovarian Res. 2023 Aug 3;16:154. doi: 10.1186/s13048-023-01223-0 (PMC10398987; doi:10.1186/s13048-023-01223-0)
Supplement: Supplementary file 4 — Additional file 4: Supplemental Table 4. The primers for real-time quantitative PCR. [file 13048_2023_1223_MOESM4_ESM.pdf]

Primers for real-time quantitative PCR

Gene	5'-3' sequence
hsa-miR-205-5p-N	GTCGTATCCAGTGCAGGGTCCGAGGTATTCGCACTGGATAC GACCAGACT
hsa-miR-205-5p-F	CGTCCTTCATTCCACCGG
hsa-miR-205-5p-R	AGTGCAGGGTCCGAGGTATT
PNPLA3-F	GAAGGCCAGGAGTCGGAAC
PNPLA3-R	TGCCTATTTTGCCGGAGATGA
MVD-F	CGTGGCATCGGTGAACAACT
MVD-R	GTGTAGGCTAGGCAGGCATA
hsa_circ_0020555-F	CGTCCTGGCACCAATTTCTG
hsa_circ_0020555-R	AATGGGCTTGAAGTGCGTG
hsa_circ_0027651-F	AGTGGGATGTCTTAGATGGTGT
hsa_circ_0027651-R	GAGGCTGCACGTTACCATTT
hsa_circ_0086809-F	TTCTTTCTCCACTCTGCCCC
hsa_circ_0086809-R	ACGGCACTATCAGGGTCTTC
hsa_circ_0118448-F	ATGAAGAGCAGCGAAGGAGA
hsa_circ_0118448-R	GGGCTTCAAGGTTGTTGGTT
UGT2B7-F	GATCCCAACAACTCATCCGCT
UGT2B7-R	CAGCAGCTCACTACAGGGAA
SAA2-F	GCTTCTTTTCGTTCCTTGGCG
SAA2-R	GCCGATGTAATTGGCTTCTCTCA
SCGB2A2-F	TGGCTGCCCCTTATTGGAGA
SCGB2A2-R	TGGCATTGTCGTCTATGAACTCT
SLC30A10-F	GCTGTGCCTGGAATTAGCAGT
SLC30A10-R	ATGTGCAGGGTGGCAATAATC
