# Supplementary material for: Integrated bioinformatics analysis elucidates granulosa cell whole-transcriptome landscape of PCOS in China
Source: J Ovarian Res. 2023 Aug 3;16:154. doi: 10.1186/s13048-023-01223-0 (PMC10398987; doi:10.1186/s13048-023-01223-0)
Supplement: Supplementary file 5 — Additional file 5: Supplemental Table 5. Antibody information. [file 13048_2023_1223_MOESM5_ESM.pdf]

Antibody information

Antibody name	Manufacturer (catalog number)	Applications (working dilution)
Anti-MVD	Proteintech (15331-1-AP)	WB (1:1000)
Anti-PNPLA3	Proteintech (11442-1-AP)	WB (1:500)
