# Supplementary material for: Integrated bioinformatics analysis elucidates granulosa cell whole-transcriptome landscape of PCOS in China
Source: J Ovarian Res. 2023 Aug 3;16:154. doi: 10.1186/s13048-023-01223-0 (PMC10398987; doi:10.1186/s13048-023-01223-0)
Supplement: Supplementary file 6 — Additional file 6: Supplemental Figure 1. The PNPLA3 mRNA levels with patients’ clinical characteristics. The correlation analysis of PNPLA3 mRNA levels with BMI (A), No. of 2PN fertilized (B), No. of day 3 good-quality embryos (C), 2PN fertilization rate (D), FSH levels (E), Estradiol (F) TT levels (G), Triglyceride (H), Total cholesterol (I), LDL-C (J), HOMA-IR (K). [file 13048_2023_1223_MOESM6_ESM.pdf]

Supplemental information

Integrated bioinformatics analysis elucidates granulosa cell whole-transcriptome landscape of PCOS in China

Qingfang Li[1,2,3], *Yimiao Sang*[1,2,3], Qingqing Chen[1,2], *Bingru Ye*[1,2], *Xiaoqian Zhou*[1,2], *Yimin Zhu*[1,2,3]

Supplemental figure 1

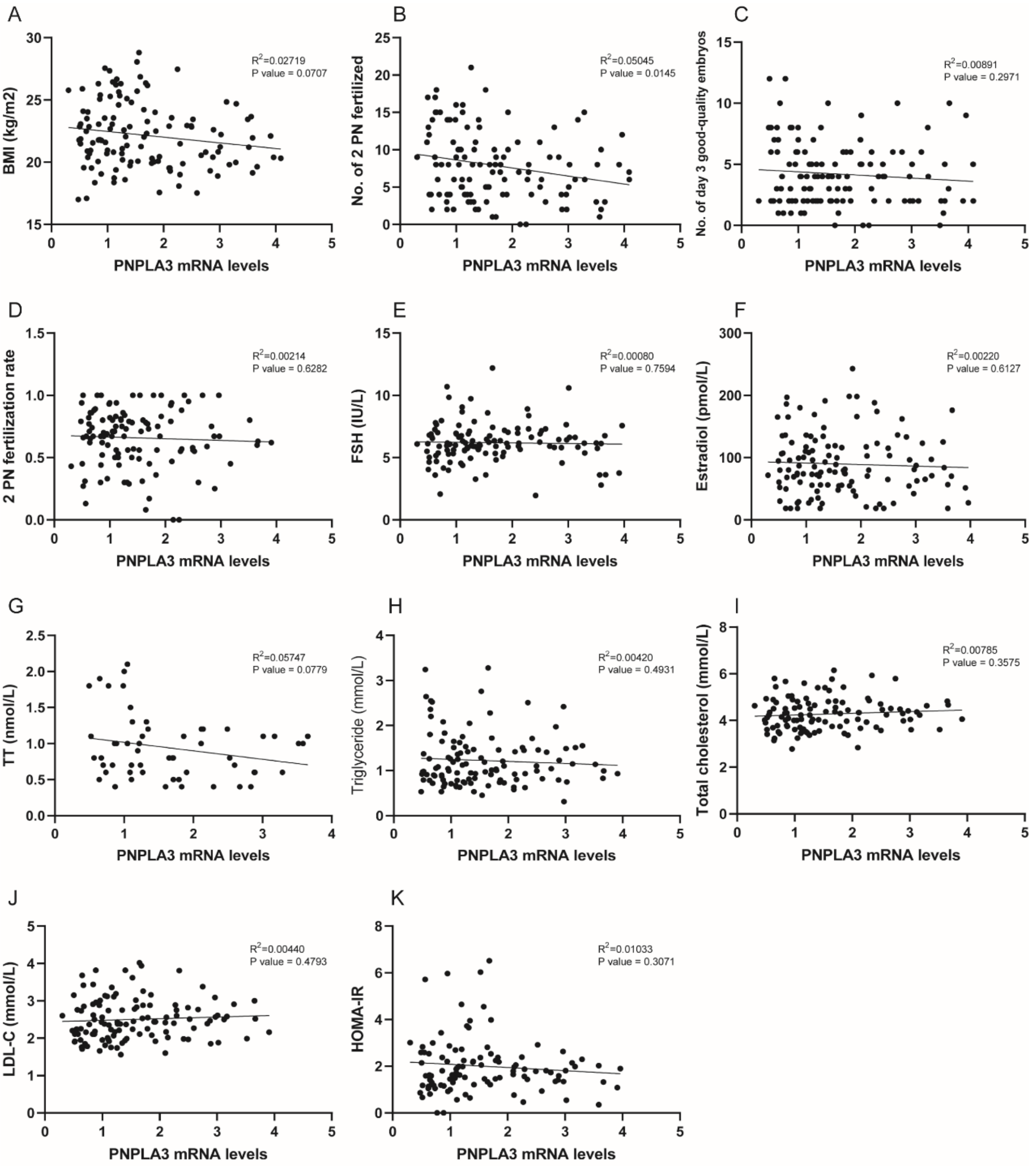

Supplemental figure 1：The PNPLA3 mRNA levels with patients' clinical characteristics. The correlation analysis of PNPLA3 mRNA levels with BMI (A), No. of 2PN fertilized (B), No. of day 3 good-quality embryos (C), 2PN fertilization rate (D), FSH levels (E), Estradiol (F) TT levels (G), Triglyceride (H), Total cholesterol (I), LDL-C (J), HOMA-IR (K).
