# Supplementary material for: Integrated bioinformatics analysis elucidates granulosa cell whole-transcriptome landscape of PCOS in China
Source: J Ovarian Res. 2023 Aug 3;16:154. doi: 10.1186/s13048-023-01223-0 (PMC10398987; doi:10.1186/s13048-023-01223-0)
Supplement: Supplementary file 7 — Additional file 7: Supplemental Figure 2. The MVD mRNA levels with patients’ clinical characteristics. The correlation analysis of MVD mRNA levels with BMI (A), AFC (B), No. of oocytes retrieved (C), No. of MII oocytes (D), No. of 2PN fertilized (E), No. of day 3 good-quality embryos (F), 2PN fertilization rate (G), FSH levels (H), P levels (I), TT levels (J), AMH levels (K), HOMA-IR (L). [file 13048_2023_1223_MOESM7_ESM.pdf]

Supplemental information

Integrated bioinformatics analysis elucidates granulosa cell whole-transcriptome landscape of PCOS in China

Qingfang Li[1,2,3]*, Yimiao Sang*[1,2,3]*,* Qingqing Chen[1,2], *Bingru Ye*[1,2]*, Xiaoqian Zhou*[1,2]*, Yimin Zhu*[1,2,3]

Supplemental figure 2

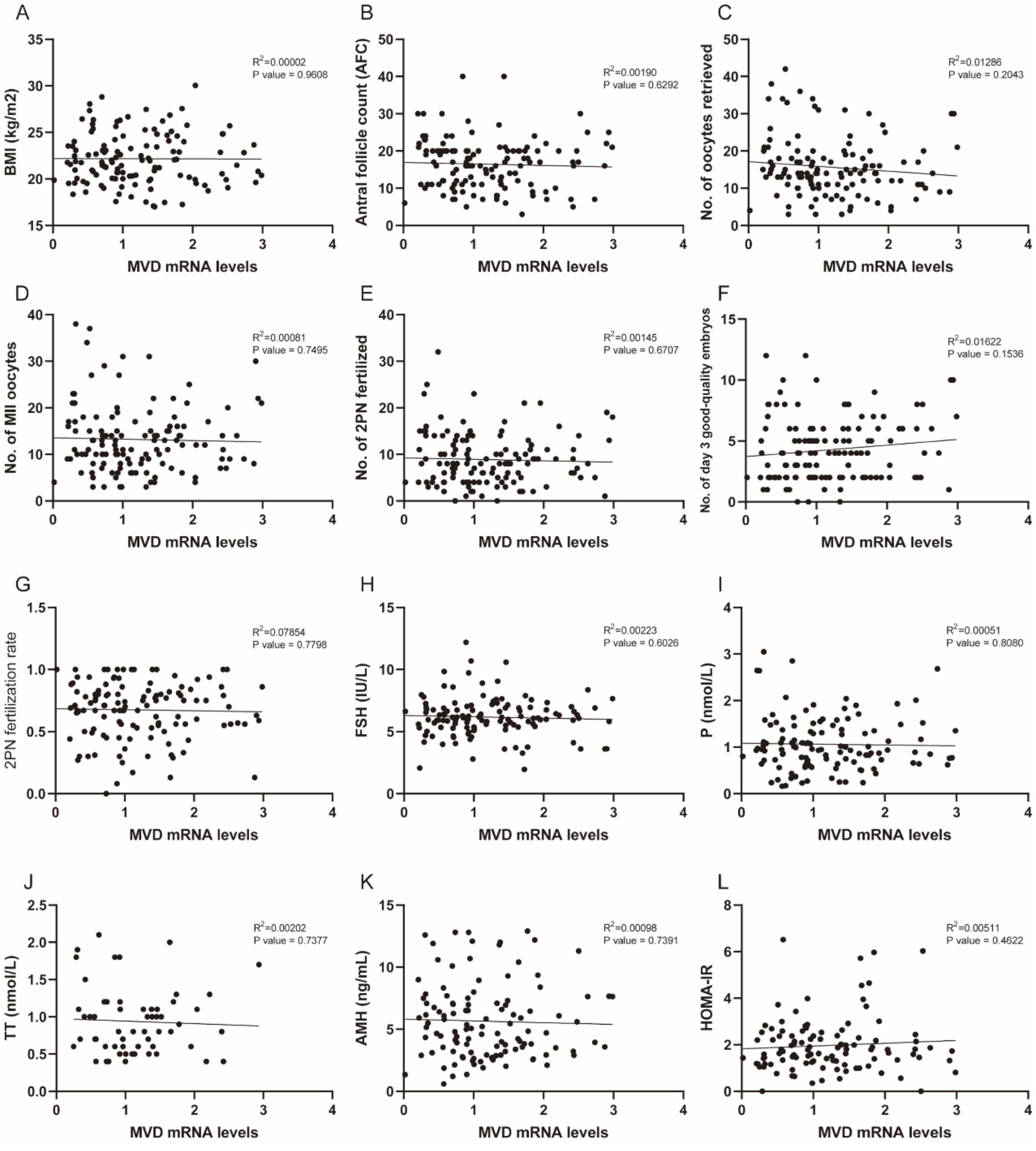

Supplemental figure 2: The MVD mRNA levels with patients' clinical characteristics. The correlation analysis of MVD mRNA levels with BMI (A), AFC (B), No. of oocytes retrieved (C), No. of MII oocytes (D), No. of 2PN fertilized (E), No. of day 3 good-quality embryos (F), 2PN fertilization rate (G), FSH levels (H), P levels (I), TT levels (J), AMH levels (K), HOMA-IR (L).
